# A Comprehensive Review of Graphitic Carbon Nitride (g-C_3_N_4_)–Metal Oxide-Based Nanocomposites: Potential for Photocatalysis and Sensing

**DOI:** 10.3390/nano12020294

**Published:** 2022-01-17

**Authors:** Amirhossein Alaghmandfard, Khashayar Ghandi

**Affiliations:** Department of Chemistry, University of Guelph, Guelph, ON N1G 2W1, Canada; aalaghma@uoguelph.ca

**Keywords:** graphitic carbon nitride, g-C_3_N_4_, metal oxide, photocatalysts, sensors, bacterial disinfection, supercapacitors

## Abstract

g-C_3_N_4_ has drawn lots of attention due to its photocatalytic activity, low-cost and facile synthesis, and interesting layered structure. However, to improve some of the properties of g-C_3_N_4_, such as photochemical stability, electrical band structure, and to decrease charge recombination rate, and towards effective light-harvesting, g-C_3_N_4_–metal oxide-based heterojunctions have been introduced. In this review, we initially discussed the preparation, modification, and physical properties of the g-C_3_N_4_ and then, we discussed the combination of g-C_3_N_4_ with various metal oxides such as TiO_2_, ZnO, FeO, Fe_2_O_3_, Fe_3_O_4_, WO_3_, SnO, SnO_2_, etc. We summarized some of their characteristic properties of these heterojunctions, their optical features, photocatalytic performance, and electrical band edge positions. This review covers recent advances, including applications in water splitting, CO_2_ reduction, and photodegradation of organic pollutants, sensors, bacterial disinfection, and supercapacitors. We show that metal oxides can improve the efficiency of the bare g-C_3_N_4_ to make the composites suitable for a wide range of applications. Finally, this review provides some perspectives, limitations, and challenges in investigation of g-C_3_N_4_–metal-oxide-based heterojunctions.

## 1. Introduction

Graphitic carbon nitride (g-C_3_N_4_) is a polymeric, visible-light-active photocatalyst with a bandgap of ~2.7 eV (~460 nm), that was introduced since 2006 [1]. g-C_3_N_4_ has become an important material in chemistry, physics and engineering because of its facile, low-cost, environmentally-friendly preparation methods with promising stability and good physicochemical properties for use in a wide range of applications [2]. Compared with other semiconductors, g-C_3_N_4_ can be easily synthesized by various methods with desirable electrical structures as well as morphologies, and high thermal stability up to 600 °C in the air [3,4].

The most common precursors used to prepare g-C_3_N_4_ are melamine, dicyandiamide, cyanamide, urea, thiourea, and ammonium thiocyanate. Among different types of carbon nitrides such as α-C_3_N_4_, β-C_3_N_4_, cubic C_3_N_4_, pseudocubic C_3_N_4_, with bandgaps of around 5.49 eV, 4.85 eV, 4.30 eV, and 4.13 eV, respectively, g-C_3_N_4_ is the most stable phase under ambient conditions [2]. In order to enhance the performance and modulate the properties of g-C_3_N_4_, researchers have proposed different methods such as doping and making heterojunction with other materials. Examples of these materials are metal oxides, metal sulfides, noble metals, and carbonaceous nanomaterials [5,6,7,8,9,10,11,12]. Among them, metal oxides are the most common ones to improve the efficiency of g-C_3_N_4_, e.g., increasing the light absorption and reducing the recombination of electrons and holes by promoting the separation of charge carriers. This is mainly due to their suitable band structures [13,14,15,16,17,18].

The g-C_3_N_4_ structure has been widely used in many applications, in particular in energy-related applications. Energy consumption to provide electricity and heat will rise to twice its current consumption by 2050, which is mainly due to industrialization, urbanization, and population growth [19,20]. The consumption of fossil fuels, such as natural gas, coal, and oil, should be decreased as their usage results in detrimental environmental impacts [2,19,20]. Two remedies are solar energy and photocatalysis [21,22,23,24]. Both require suitable semiconductors such as g-C_3_N_4_ with superior activities for different catalytic reactions, such as organic pollutants degradation, H_2_, and O_2_ generation by water splitting and CO_2_ reduction to hydrocarbon fuels [14,25,26,27,28,29]. The g-C_3_N_4_ can also be used for water disinfection and bacterial control [25,30].

**Figure 1:**
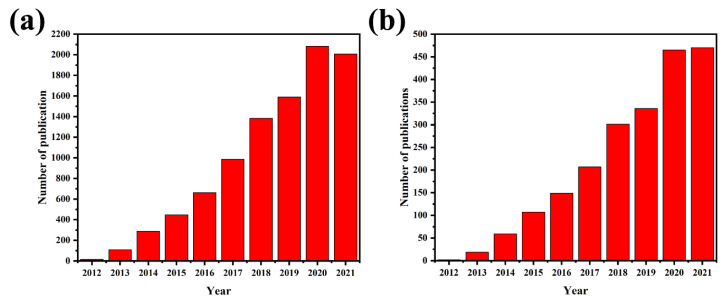
Number of annual publications (**a**) using “g-C_3_N_4_*” as a keyword since 2012, (**b**) using “g-C_3_N_4_*” with metal oxides (“TiO_2_”, “ZnO”, “WO_3_”, “Iron Oxide”, “Tin Oxide”, and other metal oxides) as keywords since 2012. Adapted from Scopus database, dated 1 October 2021.

It is evident from Figure 1 that the number of publications, collected from the Scopus database, has been growing fast from 2012 in the field of g-C_3_N_4_ and g-C_3_N_4_–metal oxide-based heterojunctions. Figure 1a shows the number of all publications on g-C_3_N_4_ since 2012, showing that this topic is among the hot research areas. It is therefore important to provide a comprehensive review of the g-C_3_N_4_–metal oxide composites. To the best of our knowledge, no publication reviews a wide range of papers to explain the applications and structure of these heterojunctions comprehensively.

This review covers the research up to 1 October 2021. We highlighted some general information about the structure and characterization of the bare g-C_3_N_4_. We also discussed some modifications such as doping to improve the g-C_3_N_4_ properties. As well, we have also summarized the research on modification of the structure and properties, to enhance the efficiency of g-C_3_N_4_ for different applications, via combining g-C_3_N_4_ with metal oxides such as TiO_2_, ZnO, iron oxide, WO_3_, and tin oxide. After reviewing g-C_3_N_4_–metal oxides, we focus on the applications of these kinds of heterojunctions. In the last part of this review article, we suggest some potential investigations for the future in this field that have not been conducted to this date to our knowledge. We also recommend some studies to better understand the nature of these heterojunctions. Most graphs in this review are reproduced by us from the data in original research papers cited, unless stated otherwise in the captions where the permission has been obtained.

## 2. Structure and Properties of g-C_3_N_4_

**Figure 2:**
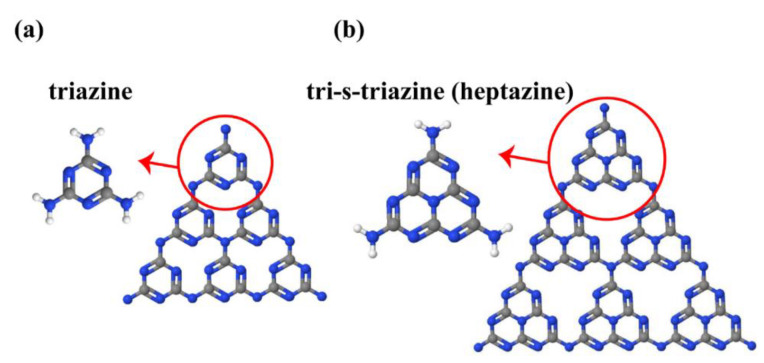
(**a**) Triazine and (**b**) tri-s-triazine (heptazine) structures of g-C_3_N_4_ (gray, blue, and white balls are carbon, nitrogen, and hydrogen, respectively).

Melamine, melam, melem, and melon are recognized as heptazine- and triazine-based molecular compounds (coplanar tri-s-triazine unit as the elementary structural motif of g-C_3_N_4_ structure) to prepare g-C_3_N_4_. As illustrated in Figure 2, triazine (C_3_N_3_) and tri-s-triazine/heptazine (C_6_N_7_) rings are the basic tectonic units of g-C_3_N_4_.

**Figure 3:**
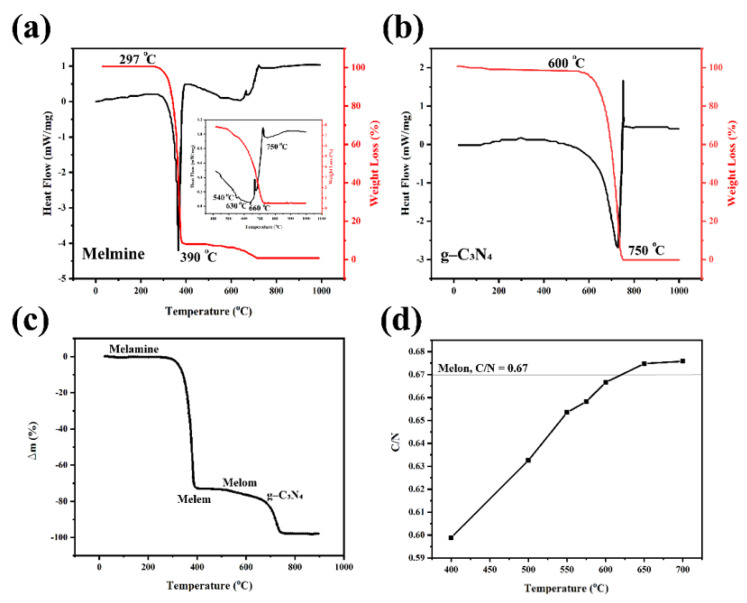
(**a**) Melamine and (**b**) g-C_3_N_4_ thermal analyses, Copyright © 2022 American Chemical society [31]; (**c**) thermogravimetric curve of annealing of melamine; (**d**) C/N values of melamine products at different temperatures, Copyright © 2022 Elsevier [32].

Yan et al. properly studied the phase transition during heating of melamine from room temperature to 1000 °C with a heating rate of 10 °C/min [31]. Figure 3a shows that melamine sublimation and thermal condensation occur at 297 to 390 °C, observed from significant endothermic differential scanning calorimetry (DSC) peak and drastic thermogravimetric analysis (TGA) weight loss. Other endothermic peaks at 545 and 630 °C are attributed to the materials’ deamination and decomposition, respectively.

Figure 3b shows the g-C_3_N_4_ thermal stability and phase transitions in an open system. This figure shows that g-C_3_N_4_ has high stability below 600 °C, which is 30 °C lower than the melamine decomposition temperature (630 °C). Beyond this temperature, g-C_3_N_4_ starts to decompose into small molecules (e.g., CO_2_ and NH_3_) [31]. The stability of g-C_3_N_4_ is higher in the semiclosed ammonia atmosphere than in the open system, mainly due to the inhibition of deamination under ammonia atmosphere. The TGA result illustrated that there is no residue at 750 °C [31]. The annealing temperature is a vital factor in the preparation and properties of the final g-C_3_N_4_ structure. Praus et al. used melamine as a precursor to synthesize g-C_3_N_4_ in an air atmosphere [32]. The TGA results, illustrated in Figure 3c, demonstrates that by heating the melamine to 400 °C melen is formed. The main reason for the weight loss at this temperature range is the elimination of ammonium. By further heating up to 600 °C, melon is obtained by melem polymerization [33]. In other words, at higher temperatures, the g-C_6_N_9_H_2_ or g-C_3_N_4.5_H is more stable than g-C_3_N_4_. Figure 3d illustrates that the C/N molar ratio is temperature dependent. The main difference in the C/N value of the synthesized g-C_3_N_4_ compared to the theoretical value of 0.75 for g-C_3_N_4_ is due to the incomplete condensation of the amino groups of melon and the low degree of polymerization. Reaction atmosphere is another factor affecting the g-C_3_N_4_ by providing defects and disordered structures. For instance, in the H_2_ atmosphere, by dicyanamide thermal condensation, more nitrogen vacancies were formed [34].

## 3. g-C_3_N_4_ Characterizations

**Figure 4:**
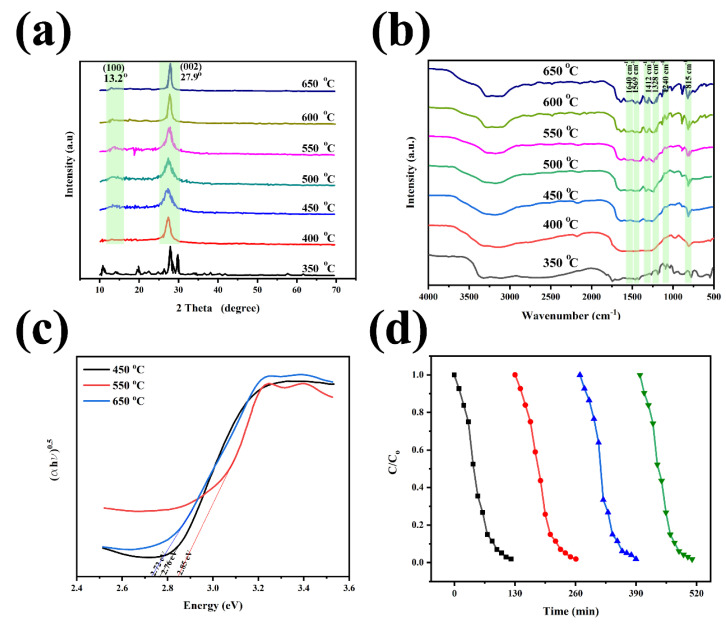
(**a**) XRD, (**b**) FTIR, (**c**) bandgaps of g-C_3_N_4_ prepared at different calcination temperatures, and (**d**) the photocatalytic recyclability degradation of dyes using prepared g-C_3_N_4_ at 550 °C [36].

The presence of X-ray diffraction (XRD) peaks at about 13 and 27° is an indication of the formation of g-C_3_N_4_, corresponding to the (002) and (100) diffraction patterns, respectively [35,36,37]. Paul et al. showed the effects of calcination temperature on the naked g-C_3_N_4_ using XRD data, Fourier transform infrared spectra (FT-IR), bandgap structure, Brunauer–Emmett–Teller (BET), and photocatalytic recyclability data [36]. Due to the polycondensation of melamine at lower than 400 °C, the crystallinity of the g-C_3_N_4_ structure is detected (Figure 4a). In contrast, Figure 4b demonstrates that no significant differences are revealed in FTIR spectra at different calcination temperatures. In the FTIR spectra, the characteristic bands with high intensity at the wavenumber about 1640, 1569, 1412, 1328, and 1240 cm^−1^ are related to the stretching modes of C=N and C–N heterocycles. Besides, the strong peaks at 815 cm^−1^ are attributed to the s-triazine units. The broad range peak between 3000 cm^−1^ and 3500 cm^−1^, is due to the N-H stretching and the remaining water molecules in the structure [35,36,37,38,39]. The BET specific surface areas were estimated to be 37.8, 73.7 and 65.6 m^2^·g^−1^ for g-C_3_N_4_ synthesized at the calcination temperature of 450, 550 and 650 °C, respectively. Calcination temperature also affects the g-C_3_N_4_ bandgap. The bandgap when calcinated at 550 °C is narrower than when calcinated at 450 and 650 °C (Figure 4c) [36,38,40]. When the temperature increases to 700 °C, a higher bandgap is observed. In other words, the higher the degree of g-C_3_N_4_ polymerization, the larger the π-plane conjugation degree of heptazine rings via N_2_ atoms, at the higher temperature. 

To assess the catalytic activity, ultraviolet-visible (UV-vis) diffuse reflectance spectrums (DRS) of the materials were determined at different temperatures [36]. As discussed earlier the results show that the bandgap of g-C_3_N_4_ prepared at 550 °C is narrower than those formed at 450 and 650 °C, so the structure prepared at the 550 °C absorbs more visible light, which can lead to the more appealing photoactivity. Moreover, the adsorption and degradation efficiencies for methylene blue dye by g-C_3_N_4_ prepared at 500 °C is 34.4% and 62.6%, which is higher than those synthesized at 450, 550, 600, and 650 °C [36]. Figure 4d reveals that the prepared g-C_3_N_4_ at a calcination temperature of 550 °C also showed good stability even after four cyclic runs. The pH, and catalyst loading are also important factors in the adsorption and degradation efficiency [36]. 

**Figure 5:**
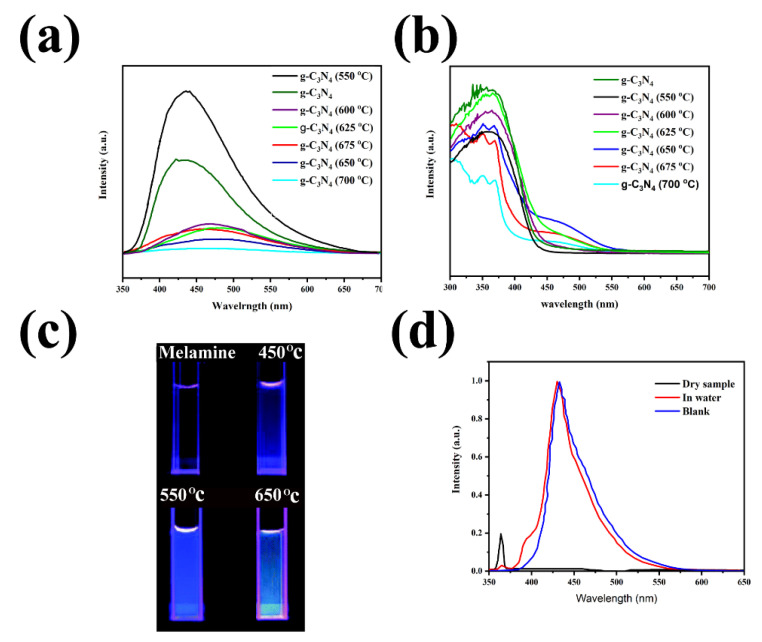
(**a**) The PL spectra under 325 nm excitation (**b**) UV-Vis spectra of g-C_3_N_4_, prepared at different temperatures, Copyright © 2022 American Chemical Society [41]; (**c**) The g-C_3_N_4_ in deionized water under 365 nm light; (**d**) the PL spectra of the g-C_3_N_4_, g-C_3_N_4_ in water and in the DI water [44].

Chen and coworkers illustrated that the PL depends on the condensation temperature (Figure 5a), showing the strong and stable emission in the range of 450–510 nm, mainly due to the π*–π, and π*–LP transitions [41]. The increase in the amount of the tri-s-triazine content at high temperature leads to the higher π states and cause orbital overlap and reduces the PL intensity. The UV-Vis spectra of the g-C_3_N_4_ at different synthesis temperatures are illustrated in Figure 5b. Like the PL emission peak, the strong absorption peaks at 450 nm and 500 nm are due to the π*–π and π*–LP transitions. Another study, conducted by Dias et al., concluded that during thermal treatment of g-C_3_N_4_, the enhanced porosity is mainly due to the creation of N vacancies and defects/holes within the nanosheets, resulting in the improvement of optoelectrical properties. Not only the treatment leads to the appearance of n → π∗ transitions, but we can also observe red shift in the absorption spectra. Moreover, it can enhance the photocatalytic activities by improving the carrier separation, reducing charge recombination, which is mainly due to the presence of trap states [42]. Furthermore, researchers also investigated the effect of the different dopants on the absorption properties of g-C_3_N_4_ [43]. These results showed the effect of temperature and dopants on the optical properties of g-C_3_N_4_. Yuan et al. showed the photograph of the melamine and g-C_3_N_4_ synthesized at different temperatures, graphically showing the color variation in the deionized water under UV light (365 nm) (Figure 5c) [44]. By increasing the synthesis temperature, the emitted color varied from blue–violet to green, and the intensity went through a maximum at 550 °C. The impact of the temperature on the PL spectra was demonstrated by the 5–10 nm blue-shift at lower temperatures, due to a less delocalized orbital resulting in a larger bandgap. Figure 5d also reveals strong PL emission spectra with a much smaller blue-shift. This research is among the rare studies investigating the effect of the environment on PL emission spectra. The g-C_3_N_4_ time-resolved PL spectra shows 5 ns electron-hole recombination at 25 °C [45]. 

## 4. g-C_3_N_4_ Preparation

Synthetic techniques for graphitic carbon nitride (g-C_3_N_4_) were first reviewed by Thomas et al. in 2008 [26]. g-C_3_N_4_ can be synthesized with various techniques such as chemical vapor deposition (CVD), solvothermal, and plasma sputtering deposition. Chemical vapor deposition to deposit g-C_3_N_4_ thin film on indium-tin-oxide (ITO), was performed by Ye et al. [27]. In this method, the mixture of thiourea and melamine was put at the bottom of a crucible with ITO substrate above it and finally transferred to the muffle furnace. CVD has several advantages and disadvantages. To be more specific, thin films prepared by CVD are cohesive in all dimensions, which is suitable for elaborately shaped pieces and helps users to fill the insides, undersides, high aspect ratio holes, etc. CVD does not require high vacuum and can deposit a wide variety of materials to prepare high purity composites. In contrast, physical vapor deposition (PVD) such as sputtering requires a high vacuum atmosphere. The drawback of CVD is that some CVD precursors are costly, and can be highly toxic, explosive, or corrosive, such as Ni(CO)_4_, B_2_H_6_, and SiCl_4_, respectively. The by-product of this method, including CO, or HF, can also be hazardous. The substrates are limited since they should tolerate high temperatures [46].

The graphitic carbon nitride nanocone arrays were grown onto the Ni-coated Si (100) substrate, using plasma sputtering deposition [47]. This method requires a vacuum chamber and a plasma source with a discharge cavity. Thermal condensation is an economical, energy-efficient approach, which has a higher chance for scaleup for commercialization. The solvothermal methods have some drawbacks such as more synthetic steps compared to the thermal condensation method; however, they can be cost-effective, low energy consumption methods with more controllable size and morphology [48,49,50]. 

**Figure 6:**
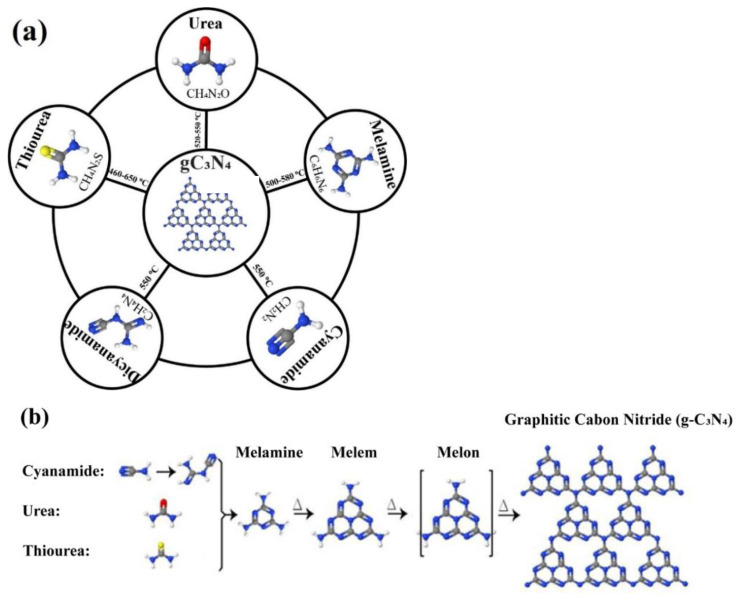
(**a**) Synthesis precursors and calcination temperature for g-C_3_N_4_ preparation; (**b**) synthesis procedure using cyanamide (dicyanamide), urea, thiourea, and melamine for g-C_3_N_4_ synthesis (gray, blue, red, yellow, and white balls are carbon, nitrogen, oxygen, sulfur, and hydrogen atoms, respectively).

Several C−N precursors such as urea, thiourea, melamine, cyanamide, and dicyanamide are used for g-C_3_N_4_ synthesis (Figure 6a). The procedure is depicted in Figure 6b [51,52,53,54,55]. Among them, cyanamide and its derivates such as dicyanamide suffer from low solubility and high cost [56]. Pham and Shin showed that urea and melamine cause poor interconnection of g-C_3_N_4_ to NiTiO_3_ since melamine provides a segregated g-C_3_N_4_ structure with no connection to the NiTiO_3_ phase, and urea makes a condensed g-C_3_N_4_ structure by releasing oxygen-containing gas during the thermal condensation [56]. Dicyandiamide and thiourea with higher reactivity towards a polymerization reaction create strong Ti−N bonds in the composite, and allow for charge carrier formation, which is important for degradation of organic contaminants [57]. The order of photodegradation ability of bisphenol A (BPA) is g-C_3_N_4_-Melamine in N_2_ atmosphere ≈ g-C_3_N_4_-dicyandiamide > g-C_3_N_4_-dicyandiamide in N_2_ atmosphere > g-C_3_N_4_-Melamine > g-C_3_N_4_-Urea ≈ g-C_3_N_4_-Urea in N_2_ atmosphere [58]. The differences between the catalytic activities are mainly due to the different preparation procedures, which can change the type, the density of active sites, the network of sp2 hybridized carbon, nitrogen, and oxygen-containing functional groups [58]. Jung et al. showed that the precursor could affect the morphology of the g-C_3_N_4_-based systems [59]. The good binding of dicyandiamide (DCDA) to ZnO nanoparticles, lead to the formation of the core-shell morphology DCDA-CNZ composite, resulting in improving the degradation of methylene blue by charge transfer [59]. In contrast, due to the weak interaction of thio and urea with ZnO, thio and urea-CNZ have a porous and segregated morphology and produce gases during the polymerization [59].

**Figure 7:**
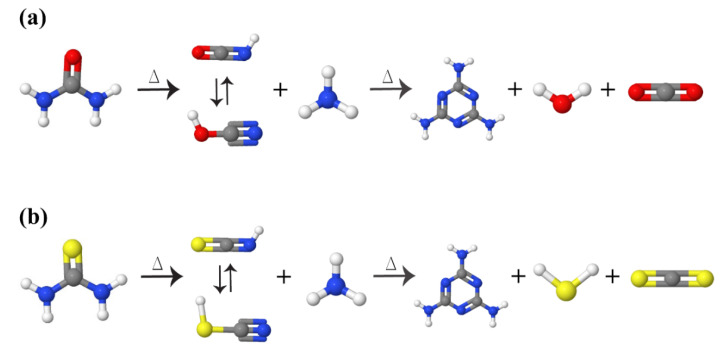
Polymerization of (**a**) urea and (**b**) thiourea into a g-C_3_N_4_ at high temperatures (gray, blue, red, yellow, and white balls are carbon, nitrogen, oxygen, sulfur, and hydrogen atoms, respectively).

As shown in Figure 6b, melamine can form from urea, thiourea, and cyanamide (dicyanamide), and then converted to tri-s-triazine (and melam) rings at ~335 and ~390 °C, respectively, and subsequently, g-C_3_N_4_ discovered from tri-s-triazine polymerization, by heating to 520 °C. Besides, the skeleton becomes prepared over 600 °C, and then beyond 700 °C, g-C_3_N_4_ is entirely decomposed into small molecules (e.g., NH_3_). Figure 7a,b shows the urea and thiourea conversion mechanism into melamine. In this reaction, oxygen atoms in the urea structure help to facilitate g-C_3_N_4_ condensation and improve stability [60]. CO_2_ and NH_3_ are the by-products of the reaction of the melamine formation from urea, which can be recycled again to urea. In the formation of the g-C_3_N_4_, the presence of a crucible lid is so important since not only it prevents the gasses from escaping, but it also provides high pressure in the synthesis atmosphere, which is necessary for the preparation of g-C_3_N_4_. In other words, a covered alumina crucible should be used during thermal analysis to avoid melamine sublimation.

During the heating reaction, the created gas bubbles act as soft templates to produce 24 nm pores in the yellow-colored graphitic carbon nitride [61]. Thiourea is used as another precursor for the g-C_3_N_4_ formation, and sulfur content improves the connectivity and packing of g-C_3_N_4_ sheets [60]. The overall conversion of urea and thiourea to melamine is endothermic. The first endothermic reaction of changing urea and thiourea to melamine is at temperatures higher than their melting temperatures, which are ~133 and ~180 °C, respectively, while at the higher temperature, melamine and heptazine were prepared in low pressure at, e.g., atmospheric pressure. To be more specific, the second reaction requires preheating to 260–280 °C to decompose urea in the presence of ammonia, passed over the activated alumina, silica gel, silica-alumina gel, or alumina gel. In order to completely form melamine, the vapors obtained from the first reaction should be maintained at ~400 °C [62]. It should also be noted that the g-C_3_N_4_ can be prepared by cyanamide and dicyanamide. Specifically, by polycondensation of cyanamide molecules and dicyandiamide, melamine was prepared at ~203 and ~234 °C, respectively [2]. 

## 5. g-C_3_N_4_ Modifications

### 5.1. Doping

The presence of heptazine ring in the g-C_3_N_4_ affects electronic structure, toxicity, and density, and is important for applications, especially as biosensors, for photocatalytic hydrogen evolution, and CO_2_ conversion. The density-functional theory (DFT) showed that the bandgap of the fully condensed g-C_3_N_4_ is lower than melem, and polymeric melon, which are 2.1 eV, 3.5 eV, and 2.6 eV, respectively [63,64,65]. As previously reported, the polymeric melon has a bandgap close to the defect containing bulk g-C_3_N_4_ [64]. There are mainly two types of experimental results to calculate the semiconductor bandgap. Specifically, the position of the conductive band (CB) and valance band (VB) can be measured by electrochemical impedance spectra (EIS), and Mott–Schottky (M–S) curve [63]. The result of both methods showed that the position of the conduction band (CB) and valance band (VB) of the g-C_3_N_4_ is about −1.3 eV and 1.4 eV, respectively [3,66]. The semiconductors’ band edges can be tuned by functionalizing, doping, compositing with other materials. Liu et al. reviewed element-doped carbonized nitrogen in detail and investigated their organic pollutants degradation applications [3]. Ai et al. showed that the bandgap value of the phosphate doped g-C_3_N_4_ decreased from 2.57 eV to 2.49 eV, 2.43 eV, and 2.41 eV by increasing the content of the P element [67]. Other researchers demonstrated that the higher O, Na, Ag, and Co content doped to the g-C_3_N_4_ structure leads to the lower bandgap value [68,69,70,71]. Li et al. investigated the effect of different ratios of Sm to g-C_3_N_4_. They illustrated that by varying the percentile molar ratios of Sm(NO_3_)_3_·5H_2_O with melamine from zero to 0.01%, 0.025% and 0.05%, the bandgap decreased from 2.63 eV to 2.57 eV, 2.50 eV, and 2.44 eV, respectively, so Sm narrowed the g-C_3_N_4_ bandgap [72]. Table 1 is illustrated the effect of different dopants on the band edge position. We will further discuss and analyze the properties and applications of different g-C_3_N_4_–metal oxides. g-C_3_N_4_ is a metal-free semiconductor, which possesses a narrow bandgap suited for visible light absorption (45% of solar energy output) [66]. To have an in-depth investigation of the optical properties of the g-C_3_N_4_, we will investigate some characteristics of the synthesized g-C_3_N_4_ such as photoluminescence (PL) and UV-vis spectra. The origin and nature of the PL emission come from three different transition pathway including π*–π, σ*—the nitride atom bridge’s lone pair (LP), and the π*–LP transition.

### 5.2. Metal Oxide-Based g-C_3_N_4_ Nanocomposite

Different types of metal oxides, such as TiO_2_, ZnO, WO_3_, iron oxide, tin oxide, etc., can improve the photocatalytic efficiency of the g-C_3_N_4_ by reducing the electrons-holes recombination and promoting the charge carriers’ separation. Consequently, metal oxide-based g-C_3_N_4_ nanocomposites can be used in different applications with enhanced electric, magnetic, and photocatalytic properties, such as H_2_ generation, CO_2_ reduction, NO oxidation, degradation of organic and inorganic dyes and other organic material, removal of toxic metal species, especially Cr (VI) from water, antibodies decontamination, solar cells, sensing, etc. [100,101,102,103,104]. In this part of the review, some metal oxide-based g-C_3_N_4_ heterojunction structures are compared with the g-C_3_N_4_.

There are five types of charge carrier separation for g-C_3_N_4_–metal oxide photocatalysts:

(1) Type I heterojunction,
(2) Type II heterojunction,
(3) Z-scheme heterojunction
(4) p-n heterojunction,
(5) Schottky junction.

Most g-C_3_N_4_–metal oxide photocatalysts show type II and Z-scheme mechanisms for charge carrier separation. In this section, we will discuss these two heterojunction types.

**Figure 8:**
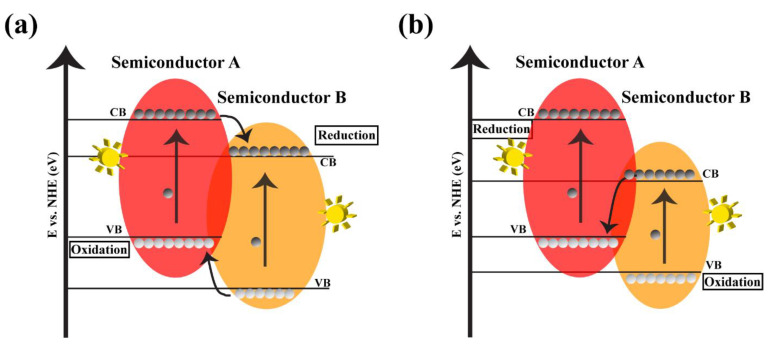
The two most common heterojunction types in g-C_3_N_4_–metal oxide photocatalysts: (**a**) type II heterojunction, (**b**) Z-scheme heterojunction (white and dark balls are holes and electrons, respectively).

In type II heterojunctions, two semiconductors are bound to form a stable heterojunction, and the position of the VB of semiconductor A is higher than that of semiconductor B. In this case, because of the difference in voltages, the photoinduced hole migrated from the VB of semiconductor B to that of semiconductor A (Figure 8a). On the other side, electrons are transferred from CB of semiconductor A to that of semiconductor B. The enhanced electrons and holes separation will reduce the rate of the recombination and promote the electrons’ lifetime. The construction of type II systems is highly desired for photocatalysis for different applications [7].

The other heterojunction type is the direct Z-scheme photocatalytic system, which was initially suggested by Bard et al. in 1995 [105]. As shown in Figure 8b, in this heterojunction type, the generated electrons on the CB of semiconductor B transfer to the VB of semiconductor A and combines with the photogenerated holes. This type of photocatalysts can be helpful for both reducing the recombination by an increase of the electrons and holes separation and improving the redox ability [7]. Even if the electrons, and holes combine and generate hν in these heterojunctions, other photogenerated carriers can replace them.

Different patterns of the migration of electrons and holes are due to the driving force of the electric field, formed by the band edge positions of different semiconductors. Depending on the electrical field direction, charge carriers start moving to reduce the energy of the system. Thus, different systems are defined based on the migration of electrons and holes in the heterojunction. The difference in each type is mainly due to the movement of electrons and holes. All types can be used in different photocatalytic activities.

#### 5.2.1. TiO_2_-g-C_3_N_4_

Among the investigated semiconductor photocatalysts, TiO_2_ has a suitable conduction band position, excellent stability, cost-effective preparation approach and is one of the most promising catalytic materials [106]. Fujishima and Honda were pioneers who researched using TiO_2_ photocatalytic behavior in 1972 [107]. To improve TiO_2_′s photocatalytic efficiency, researchers would like to reduce the bandgap of the system by doping with other elements and compositing with other compounds to absorb visible light energy [108]. To deal with TiO_2′_s limitations, researchers have been using doping elements and compositing with organic material, such as conjugated polymers and g-C_3_N_4_, since they have a narrow bandgap [108]. Boron is an effective dopant, which can improve the photocatalytic applications of TiO_2_ coupled with carbon nitride, as shown by Christoforidis and coworkers [109]. In another study conducted by this research group, TiO_2_ and carbon nitride nanosheets were synthesized by hydrothermal in-situ approach, improving the catalytic application. The mentioned materials have high porosity to ensure a high concentration of reactants in the vicinity of catalytic sites, used for CO_2_ reduction [110].

**Figure 9:**
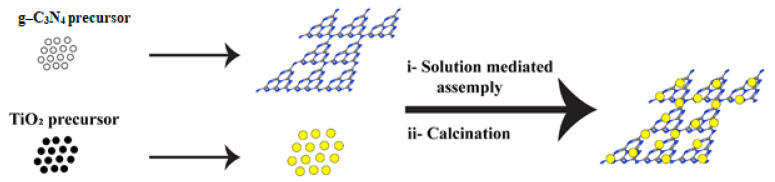
Schematic illustration of TiO_2_/g-C_3_N_4_ heterojunctions synthesis approach.

Various synthetic methods such as co-calcination, hydrothermal treatment, solvothermal, and microwave-assisted to prepare the g-C_3_N_4_-TiO_2_ heterojunction [111,112,113]. The preparation of g-C_3_N_4_-TiO_2_ heterojunction widely includes the hydrothermal and calcination method [114]. Figure 9 illustrates the schematic of the TiO_2_/g-C_3_N_4_ preparation route.

The conductive band of g-C_3_N_4_ (E_CB_ = −1.4 eV) is higher than those of anatase TiO_2_ (E_CB_ = −0.5 eV), so the proper bandgap alignment helps to reduce the electron-hole recombination by increasing their separation and boost the space charge accumulation at the interface. Recombination is defined as a process of electrons and holes annihilation. The most common type of recombination is known as radiative recombination, which occurs when electrons and holes in a conductive and valance band, respectively, recombine and emit a photon. As discussed in the following sections, the presence of electrons and holes will assist us in different stages of photocatalytic activities. So, suppressing the charge recombination is crucial in the photocatalytic applications of semiconductors and heterojunctions. At the end of section three, we talked about how g-C_3_N_4_-based heterojunction separates the position of the electrons and holes. The charge carrier separation makes electron-holes recombination less favorable, so we can use these heterojunctions for photocatalytic activities.

**Figure 10:**
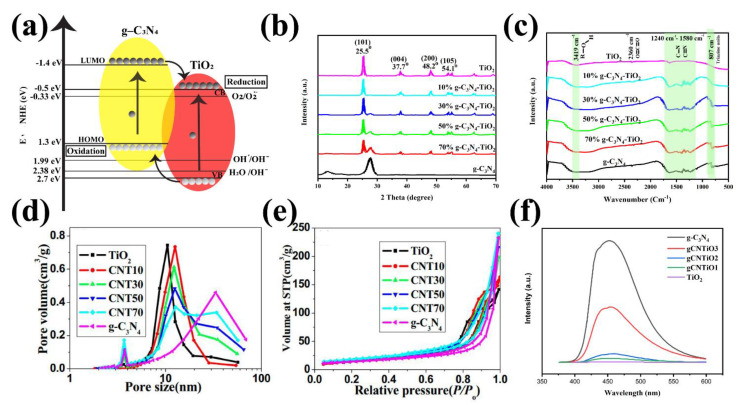
(**a**) Proposed mechanism for charge transfer of type II of the g-C_3_N_4_-TiO_2_ heterojunction interface under visible light irradiation; (**b**) XRD patterns; (**c**) FT-IR spectra of the g-C_3_N_4_-TiO_2_ heterojunction; (**d**) the pore size distribution curves; (**e**) UV-vis spectra of the prepared TiO_2_, g-C_3_N_4_, and TiO_2_- g-C_3_N_4_ composites, Copyright © 2022 Elsevier [120]; (**f**) PL emission spectrum of TiO_2_, g-C_3_N_4_ -TiO_2_, and g-C_3_N_4_ products, Copyright 2019 © American Chemical Society [122].

Figure 10a illustrates the conduction, valance, and bandgap position of the g-C_3_N_4_ and TiO_2_ and reveals that the g-C_3_N_4_-TiO_2_ structure improves the photo-induced electrons flow from the g-C_3_N_4_ conductive band to that of TiO_2_, which promotes the photoelectrical ability of the final composite. Thus, the photogenerated electrons tend to accumulate in the TiO_2_ conductive band since the conductive band of TiO_2_ is more positive than that of g-C_3_N_4_, (Figure 10a). In contrast, the holes transfer in an inverse way, which can provide type II heterojunction [112,115]. Due to the high recombination barrier, the heterojunction provides a high interfacial area for facilitating carrier transformation and separation to suppress the electron-hole recombination to improve the photocatalytic activity.

Kočí et al. successfully deposited TiO_2_ on the g-C_3_N_4_ surface by hydrothermal approach followed by calcination processing [116]. In this research, the mixture of TiO_2_ and g-C_3_N_4_, prepared by thermal hydrolysis and polycondensation from melamine, respectively, should be mixed in distilled water for 16 h in an air atmosphere dried at 60 °C. Finally, the dried sample should be maintained at 450 °C in a covered crucible with a heating ramp of 15 °C/min in the air in a muffle furnace. The highest photocatalytic performance was obtained under UVA (λ = 365 nm) irradiation compared to other types of ultraviolet (UV) rays (UVB, and UVC), which is attributed to the high separated charge carrier. The low rate of recombination leads to the great photocurrent stability. In another work on g-C_3_N_4_-TiO_2_ heterojunction structure, Alcudia-Ramos et al. demonstrated that the prepared heterojunction has a higher photocatalytic efficiency than the individual g-C_3_N_4_ and TiO_2_ [112]. The researcher also showed that solvothermal synthesis enhances tri-s-triazine’s carbonization [117]. Miranda et al. used an impregnation method to prepare g-C_3_N_4_-TiO_2_ [118]. The presence of the g-C_3_N_4_ in the heterojunction endows the high specific area to the structure. The researchers also used the photochemical reduction method to prepared g-C_3_N_4_-TiO_2_ based nanocomposite. They also demonstrated that in the TiO_2_/g-C_3_N_4_/G composite, TiO_2_ was a semiconductor to capture visible light and also prevents g-C_3_N_4_/G stacking [119].

Several parameters affect the g-C_3_N_4_-TiO_2_ heterojunction structure. Various research works have been investigated the effect of the g-C_3_N_4′_s ratio. Wang et al. prepared this microstructure with 10%, 30%, 50%, 70% of g-C_3_N_4_, which are labeled with (x = 10, 30, 50, and 70) [110]. All the XRD results from different publications have shown the same diffraction pattern. As a case in point, the XRD pattern of g-C_3_N_4_-TiO_2_ is illustrated in Figure 10b. In the TiO_2_ diffraction pattern, the peaks at 25.5, 37.7, 48.2 and 54.1 °C are related to the (101), (004), (200) and (105) planes, respectively. Furthermore, there is no g-C_3_N_4_ characteristic peak at 13.1 °C in the g-C_3_N_4_-TiO_2_ hybrid sample, which may be attributed to the low crystallinity of g-C_3_N_4_ compared to the TiO_2_ in the microstructure. However, the peak at 27.5 is obviously observed in all samples. The more g-C_3_N_4_ ratio in the structure leads to the higher XRD intensity at 27.5 °C and the lower peak intensity at 25.5 °C [120]. TiO_2_ possesses a high crystallinity so the characteristic peak at 25.5 °C is higher than 27.5 °C. There are no obvious changes in other characteristic peaks in the composite XRD patterns. The FT-IR spectra of the g-C_3_N_4_-TiO_2_ structures are demonstrated in Figure 10c [120]. In all FT-IR analyses, stretching vibration of the Ti–O and Ti–O–Ti is observed at about 475 cm^−1^. Additionally, peaks at 3419 cm^−1^ are attributed to the absorbed moisture and hydroxyl group in the structure. The peak at 2360 cm^−1^ that appeared in all samples is mainly due to the adsorbed CO_2_. All the characteristic peaks at TiO_2_ and g-C_3_N_4_ are observed in the composite samples. The pore size distribution of the g-C_3_N_4_, TiO_2_, g-C_3_N_4_-TiO_2_ is illustrated in Figure 10d [120]. The pore size at ~3.5 nm, 10 nm, and 3.8 nm, 32 nm is due to the presence of TiO_2_, and g-C_3_N_4_, respectively. Additionally, the BET specific area of the g-C_3_N_4_-TiO_2_ hybrid structure was increased at the higher g-C_3_N_4_ content. Finally, the higher the bandgap of TiO_2_, the lesser the absorption wavelength at higher than 400 nm (Figure 10e). The g-C_3_N_4_ revealed the absorption band extending to about 430 nm, which is mainly due to the low bandgap [120]. The blue shift in the absorption spectra was also demonstrated at a higher g-C_3_N_4_ ratio in the structure. It was shown bandgaps calculation of the g-C_3_N_4_, TiO_2_, g-C_3_N_4_-TiO_2_. The results revealed that the bandgap of CNT50 samples is 2.92 eV, which is like the bulk g-C_3_N_4_ with the bandgap of 2.9 eV and narrower than the bandgap of TiO_2_ (3.20 eV). Due to the narrow bandgap and heterojunction formation, the hybrid composite not only suggested the higher generation of the electron and hole but also improved photocatalytic activity [120,121]. Li et al. synthesized g-C_3_N_4_@TiO_2_ hollow sphere nanostructure with high crystallinity [122]. In this work, researchers used the different ratios of TiO_2_ hollow sphere and melamine (1:2, 1:4, and 1:8) in the solution, which are called HS-CNTO1, HS-CNTO2, and HS-CNTO3, respectively. They demonstrated that the recombination rate of the photogenerated electron-holes decreased by introducing the TiO_2_ in the g-C_3_N_4_ structure. In all samples, the sharp PL emission peak at 455 nm can be observed. The intensity of the PL peak reduced as the g-C_3_N_4_ content ratio decreased [122]. In addition, as can be seen in Figure 10f, the electron resistance decreased when g-C_3_N_4_-TiO_2_ heterojunction was used. Additionally, stability is another vitally important factor in determining the photocatalytic activities, and it is shown that the g-C_3_N_4_-TiO_2_ heterojunction demonstrates excellent stability after three to five cycles under different circumstances [117,120,122,123].

Many factors may affect the g-C_3_N_4_-TiO_2_ heterojunction productivity and improve the photocatalytic activity by enhancing the charge carrier separation and prevent recombination [124,125,126]. Rathi and coworkers showed that the CuNi@g-C_3_N_4-_TiO_2_ nanocatalyst had a 3-fold and 5-fold higher photocatalytic activity than bare g-C_3_N_4_ and TiO_2_ nanorod for Rhodamine B degradation. Besides, the photocurrent density of the TiO_2_ nanorod, bare g-C_3_N_4_, Cu@g-C_3_N_4_, Ni@g-C_3_N_4_, and TiO_2_/CuNi@g-C_3_N_4_ is 0.108 mA/cm^2^, 0.377 mA/cm^2^, 0.530 mA/cm^2^, 0.6012 mA/cm^2^, and 0.890 mA/cm^2^, respectively. It should also be mentioned that the charge separation was promoted since the presence of Cu and Ni species [126]. In another work, researchers also prepared Ti^3+^-TiO_2_/O-g-C_3_N_4_ heterojunctions via a hydrothermal approach [125]. In this synthesis approach, 1 g g-C_3_N_4_ should disperse with titanium oxohydrides sol precursor at room temperature for 20 min, ultrasonically. The collected sample should transfer into the Teflon-lined autoclave at 160 °C for 27 h and then be washed and dried at 60 °C for 3 h. This method was used to prevent the g-C_3_N_4_ aggregation and fabricate exfoliated g-C_3_N_4_ nanosheets. The synthesized heterojunction significantly decreased the regenerated electron-hole pairs’ recombination. Additionally, the conductivity is greatly enhanced and widens the light absorption range by adding the Ti^3+^, and O. P is another element used to improve the heterojunction connection and promote photocatalytic activity by facilitating the carriers’ transfer and separation [127,128].

Various novel metal nanoparticles are leading to the improvement of photoexcited semiconductors because the surface plasmonic effect is beneficial to reduce the photogenerated electron-hole recombination, improve the efficiency of visible light absorption and photocatalytic activity. Silver, and gold nanoparticles, for instance, have high stability and good conductivity. Au nanoparticles can increase the electron concentration onto their surface and enhance and extended adsorption for catalytic activity by its surface π bond [129,130,131]. Ag nanoparticles (AgNPs) are also used to modify the g-C_3_N_4_-TiO_2_ heterojunction [132,133,134,135]. The presence of AgNPs not only promotes the visible light response due to the surface plasmon resonance (SPR) effect, but also, they can result in capturing the electrons, to separate them, and transfer them more easily. These electrons react with the absorbed O_2_ on the AgNPs modified TiO_2_@g-C_3_N_4_ to form O^−^_2_. With reference to the high specific area, electrical conductivity, and mobility, different graphene-based materials such as reduced graphene oxide [119,136]. Other materials can also be used to modified g-C_3_N_4_-TiO_2_ heterojunction to increase the specific area and separate the photo-induced electron-hole pairs to enhance photocatalytic efficiency [137,138,139].

**Figure 11:**
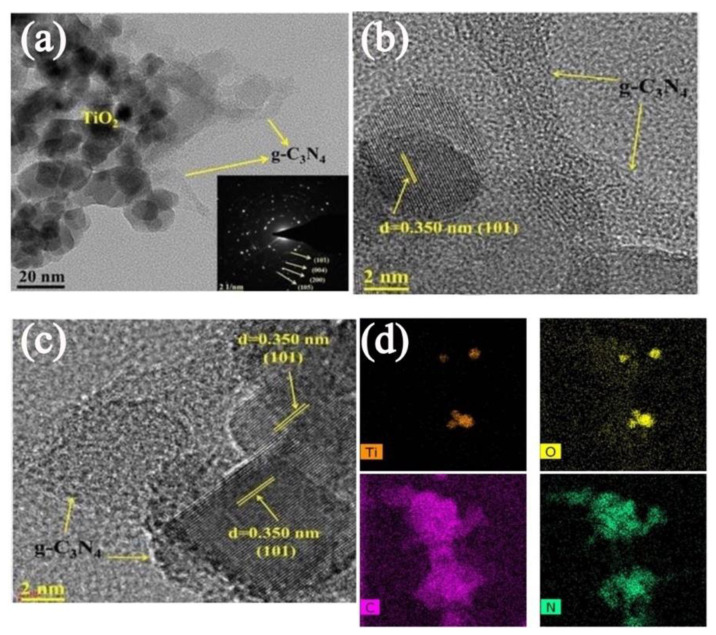
(**a**) TEM images of 5%-g-C_3_N_4_/TiO_2_ nanoparticles (inset SAED pattern); (**b**,**c**) HRTEM images of 5%-g-C_3_N_4_; (**d**) elemental mapping image of Ti, O, C, N elements of the 10%-g-C_3_N_4_/TiO_2_ composite, Copyright © 2022 Elsevier [144].

The structure and morphological evaluation of the prepared g-C_3_N_4_–metal oxide-based heterojunctions have been investigated in different research works [140,141,142,143]. Jo et al. revealed structural properties of g-C_3_N_3_-TiO_2_ heterojunctions, which are consistent with other similar composites [144]. Figure 11a showed the transmission electron microscope (TEM) micrograph and the selected area electron diffraction (SAED) pattern of 5%-g-C_3_N_4_/TiO_2_ nanoparticles. Researchers observed that g-C_3_N_4_ nanolayer uniformly is covered with TiO_2_ nanoparticles, which suggested an intimate interface between them. Besides, the inset Figure 11a showed that the circular rings corresponded to the (101), (004), (200), and (105) planes of the polycrystalline TiO_2_ nanoparticles anatase phase. A TEM image showed that the TiO_2_ nanoparticles are deposited onto the layered structure on g-C_3_N_4_. Moreover, the high-resolution transmission electron microscope (HR-TEM) images of the 5%-g-C_3_N_4_/TiO_2_ nanotube indicates the interplanar distance of 0.350 nm, attributed to the (101) plane of anatase phase of TiO_2_ (Figure 11b,c). In addition, elemental mapping analysis of the 10%-CN/TNP (Figure 11d) suggested that TiO_2_ is uniformly present onto the g-C_3_N_4_ surface. Figure 11d also revealed the presence of Ti, O, C, and N, which suggested the co-existence of both layered g-C_3_N_4_ and TiO_2_ nanotube [144].

#### 5.2.2. ZnO-g-C_3_N_4_

ZnO nanostructures such as nanosheets, nanoplates, and nanorods are semiconductors that have been used for preparing of g-C_3_N_4_-based heterojunctions. Like the TiO_2_, the ZnO bandgap is about ~3.2 eV with E_CB_ and E_VB_ of about 2.7 eV and −0.5 eV, respectively [145]. We showed that the ZnO nanostructure’s size, shape, and order could be tuned by an interplay of magnetic and gravity forces [146]. We also demonstrated the enhanced microbial detection capability when the synthesis was conducted under these external forces by changing the materials’ electrical resistance based on surface interactions. Because of low-cost of the preparation, having a large surface area, high aspect ratio, proper bandgap energy, minimal toxicity, and good stability, ZnO nanostructures have captured considerable researchers’ attention [147,148]. However, ZnO suffers from minimal light absorption (5% of the ultraviolet spectrum of the sun energy). Additionally, a high electron-hole recombination rate is another undesirable factor of ZnO for photocatalytic applications.

**Figure 12:**
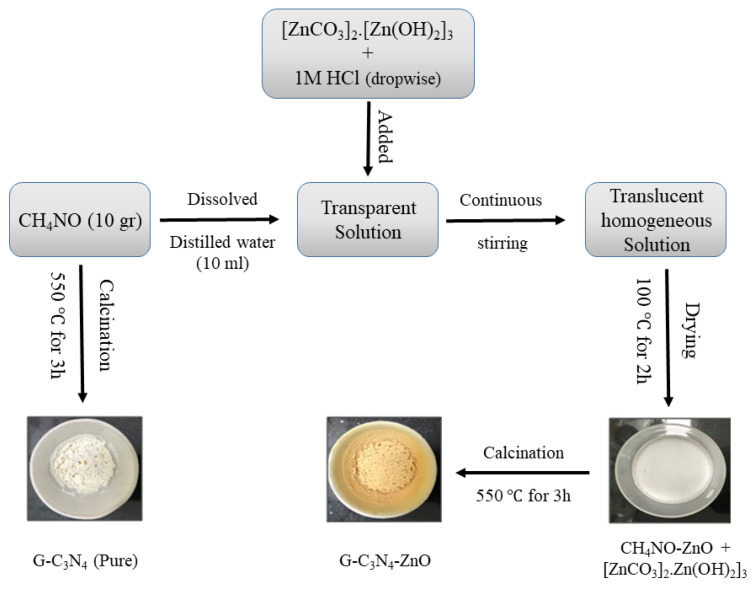
Schematic of the g-C_3_N_4_−ZnO heterojunction synthesis approach, reproduced from Reference [158].

To deal with these problems the heterojunction of ZnO with g-C_3_N_4_ might be an option for different photocatalytic applications because, coupling g-C_3_N_4_ with ZnO nanostructures can improve charge migration, separation and prevent electron-hole recombination. Researchers have reported various methods for preparing the g-C_3_N_4_-ZnO structure, such as hydrothermal, solvothermal, atomic layer deposition, etc. [149,150,151,152,153]. Jung et al. synthesized g-C_3_N_4_-ZnO with various thermal treatment and condensation temperatures (T = 350, 400, 450, 500 °C). The BET specific area and ZnO crystallite size of the prepared Z-scheme g-C_3_N_4_-ZnO structure is decreased by increasing the thermal treatment temperature [154]. Other researchers who designed g-C_3_N_4_-ZnO via thermal treatment announced that the optimal amount of the g-C_3_N_4_ content in the composite is 5.0 wt.% [155]. Melamine and ZnCl_2_ should be vigorously stirred for 20 min in a 250 mL beaker, then, Na_2_CO_3_ is added dropwise into the suspension and stirred magnetically for 30 min, and finally, dried at 60 °C for 30 min. The product was placed into a crucible with a cover to prevent from the volatilization of melamine and heated at 500 °C for 2 h at a rate of 10 °C/min. The g-C_3_N_4_/ZnO photocatalysts were obtained after deamination treatment at 520 °C for 2 h. The effect of different g-C_3_N_4_ precursors such as dicyandiamide (DCDA), urea, and thiourea on the g-C_3_N_4_ and ZnO interaction and structural morphology was investigated in another work [156]. The excellent interaction of the DCDA-ZnO results in the perfect Z-scheme charge transfer core-shell structure with the ZnO core and shell of the g-C_3_N_4_, and the low electron density of the PL emission resulting in promoting the efficiency of the Methylene Blue (MB) photocatalytic degradation. If the interaction between the precursors, such as urea and thiol, and ZnO is weak, the porous, segregated morphology is obtained. Hydrothermal method to prepare g-C_3_N_4_-ZnO heterojunction [149] not only is a low-cost preparation but also Zhang et al. revealed that it could detect nine pesticide residues in four different samples simultaneously. The solvothermal synthesis method of the preparation of the g-C_3_N_4_-ZnO modified the TiO_2_ nanotube arrays by using the ethylene glycol solution is reported by Mohammadi et al. [150]. Zhang and coworkers synthesized the g-C_3_N_4_-ZnO heterojunction composite in the metal ion-containing ionic liquid’s presence by the solvothermal method [156]. g-C_3_N_4_ was added to the ZnCl_4_ in an ethanol solution, sonicated, and mixed with NaOH. Then, the mixture should be placed in a 25 mL Teflon-sealed autoclave and maintained at 160 °C for 24 h. After washing with distilled water and absolute ethanol, the dried g-C_3_N_4_-ZnO powder was provided. The strong interaction between g-C_3_N_4_ and ZnO result in the higher migration of the generated electrons and slower recombination rate was prepared. They revealed that the power conversion of the structure composed by the solvothermal approach compared to the pure TiO_2_ nanotube arrays increases from 1.04% to 2.45%. Besides, the uniform type II heterojunction between g-C_3_N_4_ and ZnO can also be synthesized by atomic layer deposition (ALD) [151]. The mentioned heterojunction composite, synthesized by the ALD method, possessed a stable dispersion of g-C_3_N_4_ powder in the reactor, which prevented the charge carrier recombination. Mechanochemistry (mechanical milling) is also employed to prepare g-C_3_N_4_-ZnO composite [157]. This method provided significant photocatalytic stability and enhanced the composite’s photocatalytic activity, which was 3-fold higher than the bulk g-C_3_N_4_ because of the strong interaction with ZnO. Figure 12 illustrates the schematic of the ZnO/g-C_3_N_4_ preparation method.

**Figure 13:**
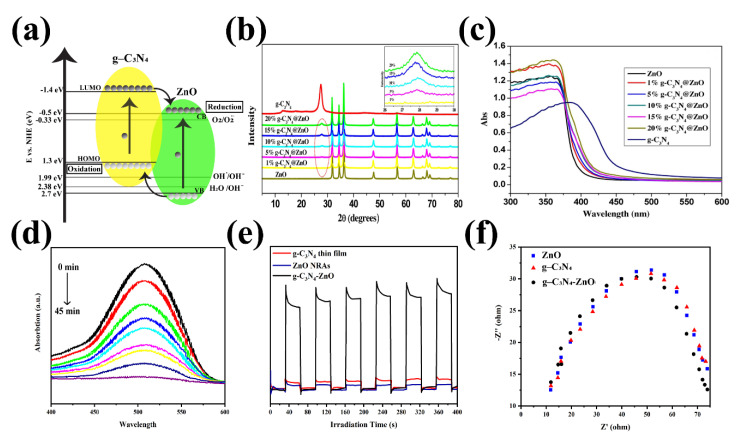
(**a**) Proposed mechanism for charge transfer of type II of the g-C_3_N_4_-ZnO heterojunction interface under visible light irradiation; (**b**) XRD patterns of ZnO, g-C_3_N_4_, and g-C_3_N_4_@ZnO samples; (**c**) UV-vis absorption spectra of ZnO, g-C_3_N_4_, and g-C_3_N_4_@ZnO composites, Copyright © 2022 Elsevier [164]; (**d**) Malachite Green (MG) photocatalytic activity of the g-C_3_N_4_/ZnO photocatalyst, Copyright © 2022 Elsevier [165]; (**e**) the pure ZnO NRAs, g-C_3_N_4_ thin film, and g-C_3_N_4_/ZnO heterostructure photocurrent response to a light on-off under visible light illumination (≥420 nm), Copyright © 2022 Elsevier [159]; (**f**) Nyquist plots of the ZnO, g-C_3_N_4_, and g-C_3_N_4_/ZnO catalysts. Reproduced from Reference [160].

Introducing the ZnO to the g-C_3_N_4_ is proved that this structure can enhance photocatalytic performance such as charge transfer and separation and decrease the photogenerated carriers’ recombination (Figure 13a) [159,160,161,162,163]. In recent years, several types of research have been worked on the characterization of these heterojunctions. For example, Wang et al. demonstrated the eight XRD characteristic peaks for the pure Zn in the g-C_3_N_4_-ZnO sample, shown in Figure 13b [164]. The reduction in the g-C_3_N_4_ content results in a decrease in the intensity of its two prominent characteristic peaks. Besides, they also revealed the FTIR analysis of the synthesized composites. The g-C_3_N_4_-ZnO composites’ FTIR peaks show the main peaks of the bulk g-C_3_N_4_, which are shifted to the lower wavenumber, and this is because of the low strength of the characteristic bonds. The FTIR peaks of the g-C_3_N_4_-ZnO heterojunction are also similar to those of the main peaks of the g-C_3_N_4_ wavenumber. The indication announces a chemical bond in the heterojunction between g-C_3_N_4_ and ZnO so that this structure will improve the charge transfer and photocatalytic efficiency. Moreover, the UV-vis DRS spectra of the g-C_3_N_4_, ZnO, and g-C_3_N_4_-ZnO heterojunction with the different g-C_3_N_4_ ratio is illustrated in Figure 13c [164]. The absorption edge of the g-C_3_N_4_ is about 460 nm, attributed to the bandgap of 2.69 eV. Besides, the absorption wavelength of the pure ZnO appeared at 396 nm showing the bandgap of 3.13 eV. Finally, the g-C_3_N_4_-ZnO revealed the redshift for the higher g-C_3_N_4_ content, which is extended to the visible-light region. The intense visible light absorption is another significant indication of the strong chemical interaction and more electron-hole generation leading to the photocatalytic activity’s improvement [164]. The high photocatalytic activity of the mentioned structure is mainly used to degrade of dyes such as malachite green (MG). After the reaction of the photocatalyst with MG, the photocatalytic rate in 0, 5, 10, 15, 20, 25, 30, 35 and 45 min was measured (Figure 13d) [165]. A similar analysis was conducted on other dye types, such as Rhodamine-B (Rh–B), Congo red (Con-R), and Red ink (RI) solution. The results show the degradation efficiency after 45 min in the presence of the g-C_3_N_4_ was determined ~97.24%, 82.37%, 70.05% and 46.99% for MG, Rh-B, Con-R, and RI’s degradation, respectively [165]. Figure 13e demonstrated the photoinduced charge transfer capability of g-C_3_N_4_-ZnO nanorod arrays. The photocurrent density of the prepared materials was generated and increased under visible light irradiation. However, the photocurrent density decreased as the illumination was stopped. It was also revealed that the g-C_3_N_4_-ZnO produced the most photocurrent density compared to the bare ZnO and g-C_3_N_4_, showing excellent charge transfer and separation. The charge transfer resistance is depicted in Figure 13f, shown impedance spectroscopy (EIS) Nyquist plots [160]. The arc radius in EIS spectra corresponds to the resistance of the interface layer at the photocatalyst surface. As observed in Figure 13f, the smaller arc radius is shown for g-C_3_N_4_-ZnO than that of g-C_3_N_4_ and ZnO. To be more specific, the smaller arc radius leads to the lower charge transfer resistance, resulting in the improved photogenerated transformation at the interface. PL spectra announce the photocurrent recombination, separation, and migration rate [160]. The ZnO PL emission wavelengths under the excitation wavelength of 350 nm were low-intensity emissions at 445 nm and 600 nm with low intensity. Besides, the emission spectra of the g-C_3_N_4_ and g-C_3_N_4_-ZnO heterojunction under the same excitation wavelength are 452 nm and 506 nm, respectively. Moreover, the higher PL intensity of the composite than that of g-C_3_N_4_ and ZnO reveals a more recombination rate of photogenerated charge carriers via the Z-scheme pathway. One of the most critical factors in photocatalytic efficiency is the catalyst’s lifetime. Recyclability of g-C_3_N_4_-ZnO is obviously illustrated by the negligible loss of the photocatalytic activity after the five cycles resulting in good stability of the g-C_3_N_4_-ZnO photocatalyst [166,167,168].

The doping of the heterojunction is a method to change and tune the properties of the bare g-C_3_N_4_-ZnO heterojunction. The metal elements are one of the excellent candidates used as dopants in the g-C_3_N_4_-ZnO structure. Ahmad et al. illustrated the effect of different metal dopants on photocatalytic efficiencies [169]. Ag-doped g-C_3_N_4_-ZnO showed the highest specific area compared to the pure ZnO, g-C_3_N_4_, and Al, Mg, Ni, Cu-doped heterojunctions. The redshift is observed for UV-vis absorption for the metal-doped g-C_3_N_4_-ZnO composite compared to the pure ZnO, revealing the more visible light absorption, and inducing a higher charge carrier generation rate [169]. The bandgap of the ZnO, g-C_3_N_4_, Al, Mg, Ni, Cu, and Ag-doped g-C_3_N_4_-ZnO, which is determined by the Tauc’s plots, is 3.23 eV, 2.66 eV, 3.15 eV, 3.08 eV, 3.06 eV, 3.0 eV and 3.05 eV, respectively. The PL spectra of these doped composites demonstrated the three emissions at 391 nm, 456–466 nm, and 550 nm, which are attributed to the free exciton transition or recombination process, bandgap recombination of photoinduced charge carriers, and oxygen vacancies [169]. The photocurrent of the metal-doped structure is higher than that of the pure ZnO and g-C_3_N_4_. The Cu-doped composite showed the lower electron transition resistance among all composites. It was concluded that the synergistic impact of Cu-doped g-C_3_N_4_-ZnO, promotes electron mobility and separation efficiency [169]. Photocatalytic activity of the Mg-doped composite is higher than that of ZnO, g-C_3_N_4_, and g-C_3_N_4_-ZnO structure. Mg is one of the ideal candidates for charge separation in the composite structure [170]. Other kinds of metal-based materials such as K, Cr, Co, and Fe have shown excellent light absorption, increased photo-induced electron-hole generation and separation, and migration for improved photocatalytic activities [171,172,173,174]. ZnO/K@g-C_3_N_4_ had higher stability after five cycles (84%) for tetracycline removal. Doping the Cr into the g-C_3_N_4_-ZnO structure promoted photocatalytic performance [172]. 60% g-C_3_N_4_/Cr-ZnO photocatalyst had 93% degradation rate in 1.5 h, which is 3.5, 2.5, and 2-fold higher than that of 5% Cr-ZnO, bulk g-C_3_N_4_, and 60% g-C_3_N_4_-ZnO, respectively. Nitrogen is another widely important element used as a dopant for the g-C_3_N_4_-based heterojunctions [147,175,176,177]. g-C_3_N_4_ with 7 wt % of N-ZnO showed the highest photocatalytic RhB degradation with 5 and 4 times higher than those of N-ZnO, and g-C_3_N_4_, respectively [175]. g-C_3_N_4_-ZnO with N doping exhibited a 77% higher H_2_ evolution rate than that of pure carbon nitride and showed a great charge carrier transfer [147,176]. The N-doped heterojunction revealed the high photocatalytic activities for methylene blue degradation since it shows the narrower bandgap [178]. Other researchers showed the impact of carbon doping in the ZnO-g-C_3_N_4_ composites [177,179,180,181]. The Z-scheme heterojunction system containing C-doped g-C_3_N_4_ grafted on the C, N co-doped ZnO was used to improve the optical properties for enhancing the BPA organic pollutants photodegradation and hydrogen evolution reaction [177]. Oxygen and sulfur are also used to improve the efficiency of the composites [181,182].

Different g-C_3_N_4_-ZnO heterojunction-based ternary composites have been constructed by various researchers worldwide [183,184,185,186,187]. The surface plasmon resonance (SPR) effect of Ag and Au nanoparticles (NPs), resulting in the improved photocatalytic activities improve by increasing the electron-hole generation and separation, so many researchers tend to use Ag NPs in the heterojunction structures [188,189,190,191,192]. Ag NPs facilitate the migration and improve the photoinduced electron-hole pairs separation by creating close interfaces between g-C_3_N_4_ and ZnO. Besides, Ag NPs reduce the energy barrier for CO_2_ to increase the intermediate radicals on the surface of the nanocomposites [191,193]. It is also revealed that the reaction constant rate for Ag (5 mol%)/ZnO/g-C_3_N_4_ is 2.4 times higher than ZnO/g-C_3_N_4_. Ag (5 mol%)/ZnO/g-C_3_N_4_ composite extends its surface, leading to promoting the photogenerated electron-holes pairs and increasing the lifetime and stability of the charge carrier [194,195]. To show the improved photocatalytic performance with high stability, different Ag-based compounds between g-C_3_N_4_ and ZnO have been investigated [188,196,197,198,199]. In another research, the hydrothermally synthesized Ag-ZnO/S-g-C_3_N_4_, comprising ZnO NPs doped with 7% Ag with 25% Sulfurized-g-C_3_N_4_, exhibited outstanding MB photodegradation (97% in 40 min) with excellent recyclability [200]. Carbon-based materials are also used beside the g-C_3_N_4_-ZnO structure [199,201,202]. Graphene oxide (GO) is carbon-based material commonly used in the g-C_3_N_4_-ZnO heterojunction [199,203,204]. GO is a two-dimensional platform, providing a promising electron conductivity, high specific surface area, and Young’s modulus, which are useful for improving photocatalytic activities. The RhB dye degradation is about 99% for ZnO-g-C_3_N_4_-GO nanocomposites in 14 min [199]. The trinary nanocomposites provide high stability, which can be used for a wide range of environmental applications [203]. Other forms of carbon-based nanomaterials such as carbon dots (C Dots) are also used to improve the heterojunction structures’ efficiency [201,205]. The C Dots provide the facile photoinduced electrons transfer from the ZnO’s CB to the g-C_3_N_4′_s VB. The Z-scheme heterojunction structure can be used in biomedical applications for bacteria-killing and acceleration of wound healing system [201].

#### 5.2.3. Iron Oxide-g-C_3_N_4_

**Figure 14:**
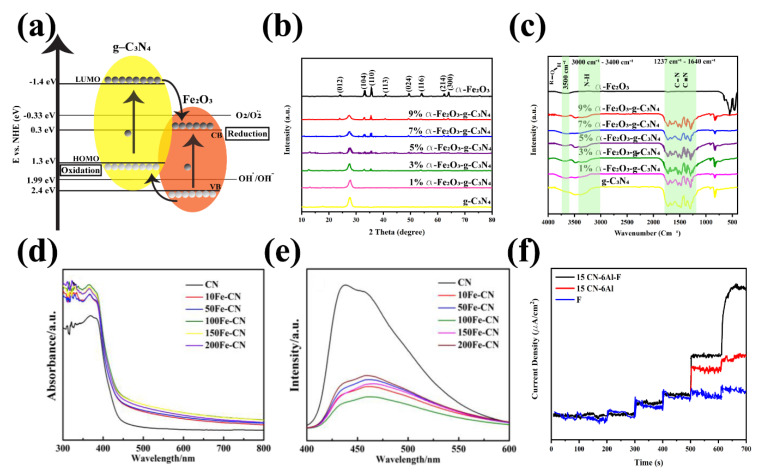
(**a**) Proposed structure of g-C_3_N_4_-FeO_x_ heterojunction interface; (**b**) XRD patterns and (**c**) FT-IR spectra of g-C_3_N_4_, α-Fe_2_O_3_, and α-Fe_2_O_3_/g-C_3_N_4_ composites, Copyright © 2022 Elsevier [220]; (**d**) UV-Vis diffuse reflection absorption spectra; (**e**) PL spectra of CN and xFe-CN samples with an excitation wavelength of 380 nm, Copyright © 2022 Elsevier [221]; (**f**) single-wavelength photocurrent response of F, 15CN-F and 15CN-6Al-F, Copyright © 2022 Elsevier [222].

Fe_x_O_y_ such as FeO, Fe_3_O_4_, and Fe_2_O_3_, not only can improve the photocatalytic performance of the g-C_3_N_4_structure due to some unique characteristics but they can also be vastly used for contaminant removal in various media [206,207,208,209]. Compared to the combination of g-C_3_N_4_ with TiO_2_ or ZnO, fewer papers have been focused on the g-C_3_N_4_-FeO_x_ heterojunctions. Xu et al. announced that the CB and VB of Fe_2_O_3_ are 0.3 eV and 2.4 eV, respectively [210]. FeO_x_ failed to show any appreciable photocatalytic activity since the improper, more positive CB edge position (Figure 14a). It is confirmed that g-C_3_N_4_/α-Fe_2_O_3_ nanocomposites obey a Z-scheme mechanism for photogenerated charge separation. Working on the g-C_3_N_4_-FeO_x_ composite was ignited by the pioneering study authored by Ye et al. [211]. They demonstrated that the efficiency of the Fe_2_O_3_/g-C_3_N_4_ photocatalysts was increased up to 1.8 times than the bulk C_3_N_4_ for the RhB degradation under visible light irradiation.

One of the most common synthetic methods to make iron oxides, especially superparamagnetic iron oxides (SPIONs), which show the highest saturation magnetization among all iron oxides while having a low magnetic coercivity, is co-precipitation method [212]. The effect of different parameters such as solution temperature, alkalinity, and stirring rate were investigated by Hosseini’s group on the suspension properties for MRI applications [213]. Iron oxides have many applications in different area such as biomedical applications, environmental application, etc. [214,215]. Wang et al. prepared g-C_3_N_4_-Fe_2_O_3_ nanocomposite via chemical co-precipitation approach [209]. They also evaluate the adsorption and desorption of seven polycyclic aromatic hydrocarbons (PAHs). Low limits of detection (LOD), excellent linearity, and recovery of the g-C_3_N_4_/Fe_3_O_4_ nanocomposites revealing their ideal candidacy for environmental applications, especially PAHs removal from water samples. In another research, the Fe_2_O_3_-g-C_3_N_4_ heterojunction was synthesized by thermal treatment in a hypoxia environment [216]. The presence of Fe_2_O_3_ in the structure will reduce the recombination rate and promote N_2_ adsorption. Some other researchers used in-situ thermal condensation to prepared g-C_3_N_4_ with iron oxides, which can be used in ciprofloxacin (CIP) degradation [217]. CIP is an antibiotic for bacteria sterilization by inhibiting the bacterial DNA. Besides, it is demonstrated that the remnant CIP in the soil can be absorbed by plants and transfer to the human body by consumption. The long-term CIP intake leads to some serious health issues [218]. The hydrothermal method is another method for preparing this composite [219]. In this method, researchers used the solution containing colloidal of the mixture of both Fe_2_O_3_ and g-C_3_N_4_. Fe_2_O_3_ could not cause the methanol yield since the low conduction band of the Fe_2_O_3_. To be more specific, Fe_2_O_3_ (5, 10, 15, 20 wt %) was added to 500 mg of g-C_3_N_4_ (in 25 mL of water), prepared by direct solid-state reaction of dicyandiamide and thiourea, to form a homogeneous mixture. The solution was transferred into the Teflon-lined stainless-steel autoclave at 150 °C for 4 h, and then washed and dried at 60 °C overnight. As a result, Duan and Mei revealed that the g-C_3_N_4_-Fe_2_O_3_ heterojunction significantly improved methanol yield from the CO_2_ photoreduction.

As mentioned above, Fe_2_O_3_ has a narrow bandgap of about 2.1 eV, making the iron oxides an excellent candidate for broad visible light absorption. Some factors, such as the high recombination rate of electron-holes, the short diffusion length of holes, and lack of sufficient conductivity, improve the need to constructing a FeO_x_-based composite, which will increase the applications of this composite. One of the most well-known FeO_x_-based composites is the g-C_3_N_4_-Fe_2_O_3_, which can adequately promote photocatalytic activities. Geng et al. illustrated the XRD and FTIR pattern of the g-C_3_N_4_-Fe_2_O_3_ composites, which are similar to the results of other publications [220]. Any significant peak shift is observed in the XRD pattern of the composites, which are indicated in Figure 14b. Additionally, the intensity of the peaks related to the (104) and (110) of α-Fe_2_O_3_ are strengthened by increasing the α-Fe_2_O_3_ content in the structure [220]. They also investigated that the FTIR characteristic peaks of the g-C_3_N_4_-Fe_2_O_3_ composites are not significantly change compared to the bare g-C_3_N_4_ and Fe_2_O_3_ (Figure 14c). As mentioned previously, the broad peak around 3500 cm^−1^ is due to the presence of moisture in the samples. The characteristic peaks at the bare Fe_2_O_3_ and g-C_3_N_4_ comprising 3000–3400 cm^−1^ and unresolved peaks from 1237 to 1640 cm^−1^ indicating N–H, C–N, and C=N in the g-C_3_N_4_, respectively [220]. Besides, the sharp peak at 811 cm^−1^ is related to the stretching vibration of the triazine units. At the same time, the peaks at 543 cm^−1^ and 469 cm^−1^ directly correspond to the Fe-O stretching vibrations. The optical studies are also depicted in Figure 14d,e, which not only showed absorption and emission of this composite but also revealed the effect composition on these characteristics features. All samples showed a promising UV absorption above 450 nm compared to the g-C_3_N_4_, helping the photocatalytic characteristics improvements (Figure 14d) [221]. The PL emission spectra showing in Figure 14e will aid us in a clearer understanding of the photophysical behavior of the prepared materials. The results showed a broad peak at the range of 440–463 nm, while the PL intensity of the Fe_x_O_y_ showed a high decrease. The decrease in the PL intensity may be due to the reduction in the luminous recombination probability, resulting in enhancing the charge separation and photocatalytic reactions. Cheng et al. also demonstrated the insignificant deactivation in about 16 h during 4 cycles showing high stability of this composition. In order to improve the photocatalytic activities, Wang and his coworkers used Al–O bridged g-C_3_N_4_-α-Fe_2_O_3_ z-scheme nanocomposites [222]. In this work, the photocurrent density with 450 nm excitation wavelength increased when researchers used the Al–O bridged 15 g-C_3_N_4_-α-Fe_2_O_3_, containing 15 mass percentage of g-C_3_N_4_ and 6 mole percent of Al to Fe. This analysis indicated in Figure 14f showed that Al–O bridged g-C_3_N_4_ facilitated the photo-generated charge transfer and separation [222].

Metastable materials that transform from one to another state over a long period of time have superior properties. Bibyite phase of iron(III) oxide (β-Fe_2_O_3_) act as its α-phase (hematite) iron(III) oxide. However, it shows a more desirable bandgap (1.8 eV) for photocatalysis. Christoforidis et al. prepared metastable β-phase Fe_2_O_3_ nanoparticles on the g-C_3_N_4_ surface by a solid-state, in-situ growth method, without the need of specialized equipment, surfactants, stabilization, or precipitating agents [223]. In this research, the β-Fe_2_O_3_ improve the photocatalytic activities by increasing the ability of light absorption in the visible region, and enhanced carriers’ separation. The hybrid β-Fe_2_O_3_/g-C_3_N_4_ nanomaterials are an excellent candidate for photodegradation since they showed higher photocatalytic activity and a promising stability [223].

There are several g-C_3_N_4_-FeO_x_-based composites with promising photocatalytic activities. The g-C_3_N_4_ coated with the Fe_x_O_y_ covered by metals will enhance the photocatalytic properties by ameliorating the photo-induced charge generation and separation. The promising LSPR effect of the Au and active sites of Pt convinced researchers to use Fe_2_O_3_/Pt/Au nanocomposite immobilized on the g-C_3_N_4_ surface as an excellent composite for hydrogen evolution [224]. As discussed in our previous papers, Au nanoparticles with strong LSPR can participate in catalytic reactions (for an example see [225]). Au NPs with a strong LSPR effect enlarge the photo-electron conversion efficiency of the photocatalyst. The researcher also investigates the CO_2_ photoreduction of g-C_3_N_4_ quantum dots-Au NPs co-modified CeO_2_/Fe_3_O_4_ micro-flowers (MFs). They showed that Au NPs promote photocatalytic activities by generating the electrons and holes and enhance carriers’ separation in CeO_2_ MFs and CN QDs in the photocatalyst [226]. Copper is another metal used in the g-C_3_N_4_-FeO_x_-based composite. Liu et al. fabricated g-C_3_N_4_/Fe_2_O_3_-Cu for electrochemical detection of glucose [227]. g-C_3_N_4_/Fe_2_O_3_-Cu composites improved the electrochemical performance for glucose detection with a LOD of 0.3 mM. They also confirmed that g-C_3_N_4_-Fe_x_O_y_ composite can be used as an electrode in sensors to measure other compounds. The effect of a multi-walled carbon nanotube was investigated by Zhang et al. [228]. They demonstrated that due to the large specific area, hydrogen bonds, π-π, and electrostatic interactions of the MWNTs@g-C_3_N_4_@Fe_2_O_3_, these 3D structures were novel magnetic solid-phase extraction sorbents for PAH with the LOD of 0.001–0.5 mgr·L^−1^. Besides, the 3D structure has a good repeatedly and recovery for 16 PAHs in the water samples. Graphene, as other carbon-based materials also used to improve the efficiency of Fe_3_O_4_/g-C_3_N_4_ composites. Wng et al. showed that the Fe_3_O_4_/graphene/S doped g-C_3_N_4_ dose of 1.0 g/L comprising 20% Fe_3_O_4_ mass fraction could completely remove Ranitidine (≤2 mg/L) in 60 min, an initial pH of 7.0 [229].

The other ternary composite used for the water splitting is g-C_3_N_4_/CeO_2_/Fe_3_O_4_ [230]. The composites showed enhanced oxygen and hydrogen evolution reaction with high current density (40 mA·cm^−2^) at the potential of 327 mV, which was greater than the bare Fe_3_O_4_ and bulk g-C_3_N_4_. The ternary composites showed excellent stability and negligible activity loss up to 14 h. the Z-scheme g-C_3_N_4_/Fe_3_O_4_ can be coupled with the CdS and was used for different antibiotics degradation [231]. The results showed the improved degradation rate to 45 times of CdS, 26 times of pure g-C_3_N_4_, and 9.5 times of CdS/g-C_3_N_4_ for the tetracycline removal. The presence of the Fe_3_O_4_ improves the photocatalytic performance and stability by an increase in the inducing and separation of the electron-hole pairs and generating more ^.^O_2_^−^ for organic pollutant degradation [231].

Other Fe_x_O_y_ compounds are also used to improve the UV-visible light absorption for different applications. NaFe_2_O_4_ is one of the most well-known FeO_x_ structures, using in the g-C_3_N_4_-based composites [232,233,234]. The Fe_3_O_4_@NiFe_2_O_4_-g-C_3_N_4_ improves photocatalytic activity up to 90% of CIP degradation by reducing the electron-hole recombination [232]. In all superparamagnetic samples, the high M_s_ assists in improvig of the recyclability and stability of the photocatalyst. The more magnetite content in the sample leads to a higher probability in the particle agglomeration, resulting in the decrease in the active sites of the and the reduction in the photocatalytic efficiencies. The fabricated g-C_3_N_4_/NiFe_2_O_4_ can also be used to degrade MB and RhB by activating H_2_O_2_ to produce the oxidizing reagent [233,234]. The magnetic properties of the NiFe_2_O_4_ (M_s_= 45 emu/g) were induced to the whole composite (M_s_= 40 emu/g) to promote repeatability and improve the photodegradation rate [233]. The other most popular compound captured researchers’ attentions are ZnFe_2_O_4_ and LaFeO_3_ [235,236,237].

#### 5.2.4. WO_3_-g-C_3_N_4_

**Figure 15:**
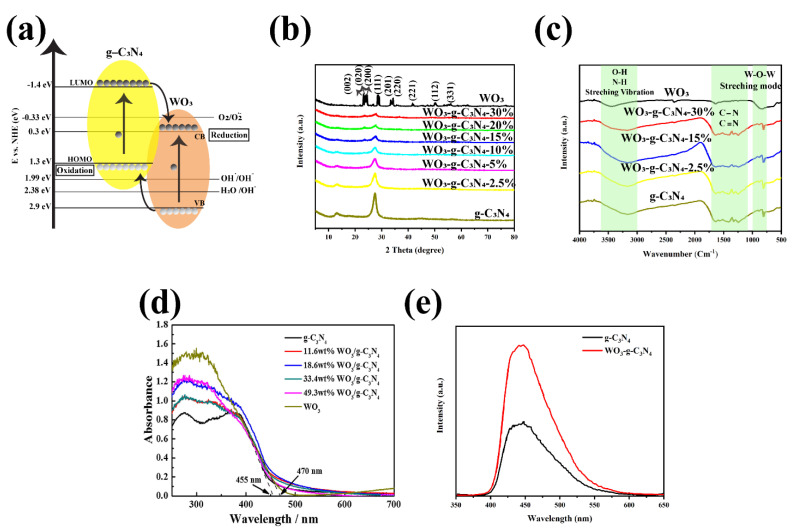
(**a**) Proposed structure of g-C_3_N_4_-WO_3_ heterojunction interface; (**b**) XRD patterns and (**c**) FT-IR spectra of obtained samples (x in WO_3_/g-C_3_N_4_ refer to the mass ratio of WO_3_ to g-C_3_N_4_), Copyright © 2022 Elsevier [251]; (**d**) UV-DRS spectra of pure g-C_3_N_4_, WO_3_ and WO_3_/g-C_3_N_4_ heterojunctionp; (**e**) PL emission spectra of g-C_3_N_4_ and 18.6 wt % WO_3_/g-C_3_N_4_ composite, Copyright © 2022 Elseviers [254].

Figure 15a demonstrated the band edge position of the WO_3_ compared to the g-C_3_N_4_. This Figure illustrated that the bandgap of the WO_3_ is 2.6 eV with VB and CB of 2.9 eV and 0.3 eV, respectively, which are more positive than that of g-C_3_N_4_ and the O_2_/O_2_^−^ potential [238,239,240]. Thus, it is approximately impossible to produce ^.^O_2_ by using the traditional type-II mechanism. Many researchers showed that the charge migration almost occurred by the Z-scheme mechanism in the binary g-C_3_N_4_-WO_3_ composite [241,242,243]. Various methods have been fabricated this binary composite. The hydrothermal approach is one of the main routes to prepare the g-C_3_N_4_-WO_3_ composite [244,245]. Zhang et al. prepared WO_3_/g-C_3_N_4_ by dissolving the WCl_6_ and ascorbic acid in ethanol and g-C_3_N_4_ followed by 5 min sonication and stirred for 20 min [244]. The uniform suspension was heat-treated at 220 °C for 12 h, and then washed several times using ethanol. The other way for the preparation of the heterojunction is the microwave irradiation technique with the direct calcining of the WO_3_ and g-C_3_N_4_ combination at 400 °C for 2 h [246]. In this method, to prepare WO_3_ by simple household microwave irradiation, tungstic acid in NaOH solution was mixed and stirred for 30 min to form a tungstic hydroxyl group [246]. Afterward, the pH of the prepared mixture was reduced to 1 by adding HCl solution, and the gel should be put into the Teflon-lined household microwave oven (2.45 GHz) for 10 min to prepared WO_3_. Finally, the mixture should be placed in an alumina crucible with a cover in a muffle furnace and heated at 400 °C for 4 h. A wet chemical process, sonochemical, in situ self-assembly, etc., are other ways to synthesize the g-C_3_N_4_- WO_3_ heterojunction [247,248,249].

g-C_3_N_4_-WO_3_ composites were characterized by various techniques. In most research papers, XRD and FTIR are the prime characteristic methods to evaluate the formation of g-C_3_N_4_-WO_3_. Researchers announced that the WO_3_/CN wt % = 10% provides the best characteristics compare to the balk g-C_3_N_4_ and WO_3_ [250]. Figure 15b shows the XRD patterns of the bulk g-C_3_N_4_, WO_3_, and g-C_3_N_4_-WO_3_, perfectly showing the impact of WO_3_ content on this characterization. There are nine distinct peaks for the as-prepared WO_3_ [247,251]. Additionally, the binary composites revealed the combination peaks of the WO_3_ and g-C_3_N_4_. The higher WO_3_ content leads to the lower peak’s intensity of the g-C_3_N_4_, corresponding to the expansion of the interlayers and g-C_3_N_4_ coverage with a WO_3_. The low intensity of the peaks at 23.5° and 36.6°, which is detectible in the sample with a high WO_3_ to g-C_3_N_4_ ratio, is related to the hexagonal-phase WO_3_ and illustrated the formation of the composite successfully. They also characterized the g-C_3_N_4_-WO_3_ composite with the FTIR spectra analysis [251]. Like other materials’ FTIR spectra, the wide peak at the range of 3000–3500 cm^−1^ in all samples is attributed to the N-H and O-H stretching vibration, and the adsorbed water molecules’ bending vibration stands at about 1630 cm^−1^ for samples (Figure 15c). The broad peak at the 750–1000 cm^−1^ corresponds to the O-W-O stretching vibrations of WO_3_ [252,253]. As a result, although the g-C_3_N_4_ characteristic FTIR peaks can be observed in the composite FTIR spectra, WO_3_ peaks are not significantly detected in the hybrid composite, which can be ascribed to the vacancies between the g-C_3_N_4_ clusters or band overlapping. Optical studies were carried out by Chai et al. on the g-C_3_N_4_ with WO_3_ structure [254]. The UV-vis DRS of the WO_3_, g-C_3_N_4_, and WO_3_-g-C_3_N_4_ composites with the WO_3_ contents is depicted in Figure 15d. The obvious absorption edges at ~470 nm and 455 nm are detected for WO_3_, g-C_3_N_4_, respectively, corresponding to the 2.64 eV and 2.73 eV. The g-C_3_N_4_-WO_3_ composites present the combination absorption features of g-C_3_N_4_ and WO_3_ [254]. Figure 15e displays the PL emission peak of the bulk g-C_3_N_4_ and 18.6 wt % WO_3_/g-C_3_N_4_ hybrid composite [254]. The result demonstrates that the intensity of the composites’ PL peak at ~440 nm is lower than that of the pure g-C_3_N_4_ [255,256,257]. The PL spectra revealed that WO_3_ apparently suppresses the photoinduced electron-hole recombination in the WO_3_/g-C_3_N_4_ composites and confirms the Z-scheme interface contact [258]. They are also revealed that the OH^–^ generating during the photocatalytic reaction leads to the higher PL intensity increases the irradiation time. Besides, the transient PL decay trace of the bare g-C_3_N_4_, WO_3_, and g-C_3_N_4_-WO_3_ composite hollow microsphere are ~ 4.46 ns, 1.62 ns, 2.23 ns, respectively [259]. The zeta potential values of the WO_3_, g-C_3_N_4_ and their binary composites announce the negatively charged surface of the samples [260]. The presence of WO_3_ in the composite changes the zeta potential from −5.7 mV to −33.1 mV, while it decreases the BET specific area from 100.97 m^2^·g^−1^ to 47.88 m^2^·g^−1^, which are high enough for promising adsorption ability and photocatalytic activities. g-C_3_N_4_-WO_3_ reveals high stability and repeatability after 4 cycles with a slight efficiency decrease for the degradation of RhB [261,262,263].

There are also some dopants and compounds used to improve the photocatalytic efficiency of the binary composites. In the z-scheme C or Pt-g-C_3_N_4_ with hydrogen treated WO_3_, the electrons from g-C_3_N_4_ and holes from WO_3_ facilitate the photogenerated charge carries generation, which will enhance the photocatalytic activities [254,264]. In other words, the C or Pt dopants help the composite to improve the light absorption and charge separation. This structure also enhances stability and repeatability. O-doped g-C_3_N_4_-WO_3_ has 4.7 times higher H_2_ performance than the bare composite [265]. The carbon vacancies and g-C_3_N_4_ oxidization resulted in the formation of the porous composites and a decrease in the composite’s surface area. The effective Si-O bridge between g-C_3_N_4_ and WO_3_ significantly promotes charge transfer and separation [266].

In addition to the doped binary composites, adding other compounds to form the ternary structure is a practical way to boost the composite characteristics. Mediators and co-catalyst such as metals (such as Ag, Au, Cu, Pt, and Sn) are currently used to improve the Z-scheme composites by facilitating the transporting and carrier capturing during the photocatalyst process [267,268,269,270,271,272]. Li et al. demonstrated the charge carrier migration between various WO_3_-Metal (Cu, Ag, Au)-g-C_3_N_4_. They showed that Cu plays the ideal candidate for photocurrent enhancement and improves the photocatalytic performance in the Z-scheme g-C_3_N_4_-WO_3_ heterojunction among different metals used in the research. In other words, the Cu-g-C_3_N_4_ and WO_3_-Cu are favorable for electron migration since they have the matched Fermi level of energy [267]. In other research, the developed WO_3_/Ag/g-C_3_N_4_ ternary composite was used for the RhB and tetracycline (TC) degradation. The results demonstrated that the improved photocatalytic activity of WO_3_/Ag/g-C_3_N_4_ is obtained due to the large contact region between g-C_3_N_4_ nanosheets and WO_3_ nanoplates. Besides, the presence of the Ag NPs in the composite with SPR effect accelerates the charges to transfer, improves the photocatalytic activity, and enhances the stability and repeatability [268,269].

Researchers also worked on the Ag/g-C_3_N_4_/WO_3_ to degrade oxytetracycline hydrochloride under visible light [273]. The 0.4 g/L of Ag/g-C_3_N_4_/WO_3_ composite demonstrated the highest photocatalytic activity, which could degrade 97.74% of oxytetracycline (10 mg/L) in 60 min. In another research, Qin et al. showed that the presence of Pt in the WO_3_-g-C_3_N_4_ composites possesses excellent photocatalytic H_2_ evolution with 1299.4 μmol under visible light, which is higher than that of WO_3_/g-C_3_N_4_/Pt and pure CN with 1119.4 μmol, and 113.2 μmol, respectively [274]. As mentioned above, g-C_3_N_4_/Au/WO_3_ as Z-scheme heterojunction displayed excellent photocurrent that can be used in photoelectrochemical immunosensing of Aflatoxin B1 in food, which can be dangerous for humans [275]. The methane formation over the WO_3_-Bt-g-C_3_N_4_ composite was 5.98, 6.74, and 25.19 times higher than that of WO_3_-g-C_3_N_4_, Bt-g-C_3_N_4_, and g-C_3_N_4_ samples, respectively [272].

Some promising properties of tungsten-based materials such as electrical, optical, magnetic, and photocatalytic activities, low-cost preparation persuade researchers to use these materials such as NiWO_4_, BaWO_4_, CuWO_4_, and BiWO_6_ [270,276,277,278]. The band structures of these compounds with g-C_3_N_4_ and WO_3_ make the CB and VB positions match each other, leading to the prolonged charge carriers’ lifetime in the form of a double Z-scheme system. The photoelectrochemical (PEC) of a double Z-scheme g-C_3_N_4_-WO_3_-Bi_2_WO_6_ system reveals the enhanced photocurrent density, reduced electron-hole recombination, resulting in the promotion of photocatalytic efficiency [276]. Other materials have been anchored in the g-C_3_N_4_-WO_3_ to form an excellent composite for different applications [279,280]. Bi-based materials are among the prime and important materials used to construct the composites for photocatalytic activities [281,282,283]. MoS_2_ and MoO_3_ are other compounds to be utilized as materials for this ternary composite [284,285]. g-C_3_N_4_/WO_3_ with TiO_2_ can also be useful for the methylene blue dye degradation, which its efficiency is about 3.1 folds higher than that of the binary counterparts of TiO_2_/WO_3_ (0.00691 min^−1^) in 120 min. The kinetic constant for different composition of this composite are in order of TWG-15% (which 15% is the g-C_3_N_4_ wt %) > TWG-10% > TWG-5% > TWG-20% > TW [286].

#### 5.2.5. Tin Oxide-g-C_3_N_4_

**Figure 16:**
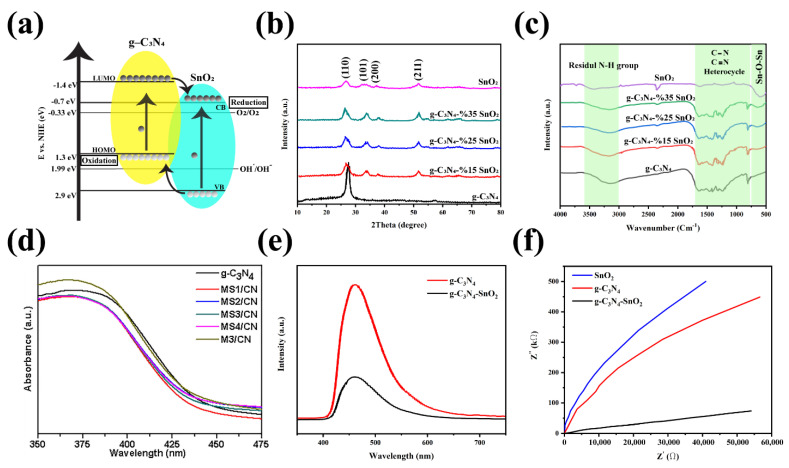
(**a**) Proposed structure of g-C_3_N_4_-SnO_2_ heterojunction interface; (**b**) XRD patterns and (**c**) FT-IR spectra of SnO_2_, graphene-like C_3_N_4_, SnO_2_/g-C_3_N_4_ composites (reproduce from Reference [295]); (**d**) UV-vis absorption spectra of g-C_3_N_4_-based samples (muscovite sheet(x)/SnO_2_/g-C_3_N_4_ (MSx/CN), which x refers to the mass of MS powder), Copyright © 2022 Elsevier [298]; (**e**) PL spectra of g-C_3_N_4_ and SnO_2_ quantum dots-g-C_3_N_4_ nanocomposite, Copyright © 2022 Elsevier [299]; (**f**) EIS Nyquist plots of the bare SnO_2_, g-C_3_N_4_, and SnO_2_-g-C_3_N_4_ structure, Copyright © 2022 Elsevier [300].

Other metal oxides widely used in g-C_3_N_4_ binary heterojunction are tin oxides. SnO (stannous oxide) and SnO_2_ (stannic oxide) are two forms of tin oxide. Figure 16a shows the band edges’ position in the tin oxide and compares it to the CB and VB of the g-C_3_N_4_. He et al. showed that the SnO_2_ has a large bandgap of about 3.6 eV, and its CB level is lower than that of g-C_3_N_4_ [287]. It is also demonstrated that these tin oxide-g-C_3_N_4_ composites mostly form the Z-scheme heterojunction [288,289]. Tin oxide can be designed with various shapes by wet and dry methods. The heterojunction is synthesized by different synthetic methods, including hydrothermal and thermal treatment [78,290,291,292]. Sadrnezhad and coworkers prepared g-C_3_N_4_-SnO_2_ with the pyrolysis of urea under microwave irradiation [293]. In this work, tin, ammonium, and urea were put into the beaker and placed in a microwave oven operating at 2.45 GHz and 900 W for 30 min. Finally, the product was washed and dried in the oven. In another work, the mesoporous SnO_2_ decorated with g-C_3_N_4_ was prepared via pulsed electrophoresis and facile water-crystallization [293]. With pulsing electrophoresis optimized parameter, the 0D g-C_3_N_4_ can be homogeneously and completely distributed inside the 1D SnO_2_. Sol-gel is also used to fabricate an efficient g-C_3_N_4_-SnO_2_ photocatalyst [294].

Several techniques have been used to confirm the formation of this composite. The XRD pattern and FTIR spectra of g-C_3_N_4_, SnO_2_, and SnO_2_-g-C_3_N_4_, are depicted in Figure 16b,c [295]. SnO_2_ does not change during the preparation of the final structure, which can be seen from the insignificant differences between the pure SnO_2_ and the SnO_2_-g-C_3_N_4_ XRD pattern in Figure 16b. The characteristic peaks in SnO_2_ revealed the (110), (101), (200), and (211) planes of a tetragonal rutile-like structure. Other small peaks, which are not mentioned in Figure 16b, are related to the (220), (310), (301), (202), and (321). As shown in Figure 16b, the (002) crystal plane peak of g-C_3_N_4_ overlapped with the (110) crystal plane peak of SnO_2_. Additionally, the Sn–O–Sn anti-symmetric stretching vibration between 400 cm^−1^ and 700 cm^−1^ was demonstrated in Reference [295] (Figure 16c). The red-shift peak at 597 cm^−1^ was a good indication of the formation of SnO_2_-g-C_3_N_4_ heterojunction [296,297]. The presence of SnO_2_ in the structure suppresses the electron-hole recombination rate. Peng et al. demonstrated the negligible differences and slight blue shift of g-C_3_N_4_-muscovite sheet/SnO_2_ structure, compared to the absorption edges of g-C_3_N_4_ (Figure 16d) [298]. Introducing the muscovite sheets does not affect the composites’ absorption, and g-C_3_N_4_-muscovite sheet/SnO_2_ absorption spectra reveal the potential photocatalytic applications under visible light. They also showed that the g-C_3_N_4_-muscovite sheet/SnO_2_ Cement has an excellent photocatalytic activity for RhB stains and isopropyl alcohol photodegradation. Due to the low electron-hole recombination probability of the SnO_2_-g-C_3_N_4_, the intensive PL intensity was observed in Figure 16e [298,299]. The faster interfacial charge-transfer and lower resistance, results in the Nyquist plot diameter decrease compared to SnO_2_ and g-C_3_N_4_, shown in Figure 16f [300]. This is therefore a promising photocatalyst. The properties and morphology of this composite change when the relative content of g-C_3_N_4_ varies in the heterojunction [301,302]. Chen et al. showed that the g-C_3_N_4_ mass ratio of 72% provides the highest photocatalytic performance, which is 17 times higher than bulk g-C_3_N_4_ [303]. SnO_2_-g-C_3_N_4_ heterojunction reveals notable stability and recyclability [18]. In addition to robust photocatalytic activities, this composite has a noticeable photoelectrochemical performance under visible light. Doping different elements, such as Sb, S, B, and P, is one of the main approaches to improve the performance of the SnO_2_-g-C_3_N_4_, which can modify the band edges [304,305,306].

Doping is a method to improve the efficiency of SnO_x_-g-C_3_N_4_ heterojunctions, which are used in different applications. SnO_2_/g-C_3_N_4_ sensor doped with Ni shows LOD about 1.38 ppb and has high stability, promising selectivity, rapid response and recovery time, and great resistivity against humidity [307]. Besides, researchers used made Ce doped SnO_2_/g-C_3_N_4_ to make capacitors with a specific capacity of <274 F/g. A supercapacitor with energy and power densities of 39.3 W h kg^−1^ and 7425 W kg^−1^, respectively, was made by using Ce-SnO_2_/g-C_3_N_4_/Activated Carbon. The supercapacitor device exhibited retention of 84.2% after completing 5000 cycles [308].

Additionally, several researchers used the SnO_2_-g-C_3_N_4_ based ternary composites to increase the applications of these kinds of composites [309,310]. The plasmonic Au-SnO_2_-g-C_3_N_4_ photocatalyst was fabricated for H_2_ evolution and degradation of the organic pollutant, which is commonly used due to the outstanding photocatalytic activities and significant stability (remain unchanged after 5 h in 5 cycles) [309,311]. In this ternary heterojunction, the presence of the plasmonic Au and g-C_3_N_4_ offer enhanced photogenerated electrons to the structure. SiO_2_ is another compound used besides the SnO_2_-g-C_3_N_4_ system for pollution treatment applications [312]. As mentioned above, TiO_2_ has promising properties that aid us in promoting the properties of this composite [313,314]. This ternary composite also demonstrated the perfect antibacterial activity for the degradation of *E. coli* bacteria, probably due to the interface between g-C_3_N_4_-SnO_2_ and TiO_2_ and lower charge recombination rate [314]. SnO_2_/chitosan/g-C_3_N_4_ nanocomposite has been used as an Electrochemiluminescence aptasensor to improve lincomycin detection [315]. Ali et al. also showed the cost-effective prepared g-C_3_N_4_/rGO/SnO_2_ nanocomposite for RhB degradation. The optimal amount of this nanocomposite reveals an increased RhB degradation efficiency [316].

As a result, the SnO_2_-g-C_3_N_4_ structure can be used in a wide range of applications. The exploitation of new clean energy instead of fossil fuels is of great interest to many since the fossil energy is exhausted. As a case in point, water splitting is a perfect example of this clean energy generation, which is widely applied by the g-C_3_N_4_-SnO_2_ [317,318]. Besides, in order to reduce environmental pollution, the demand for electric transports has been increased. Thus, it is essential to use the rechargeable batteries such as lithium-ion batteries (LIBs) and improve lithium storage capacity. Specifically, g-C_3_N_4_ enables SnO_2_ anode to enhance the Li storage in these batteries [319,320]. This heterojunction is also used to detect different compounds. Cao et al. showed that SnO_2_/g-C_3_N_4_ composite promoted the sensitivity and selectivity in ethanol gas-sensing applications [321]. The degradation of the inorganic pollutant is another application of this heterojunction [322,323]. The emission of nitrogen dioxide (NO_2_) and nitric oxide (NO) causing some environmental issues is one of the biggest challenges among several researchers, solved by Zou and coworkers by using SnO_2_-g-C_3_N_4_ photocatalysts using visible-light irradiation under 30 min [287]. SnO_2_-g-C_3_N_4_ is also used to decomposed Ammonium Perchlorate (AP), a toxic inorganic material [324].

#### 5.2.6. Other Metal Oxides

Other kinds of metal oxides such as V_2_O_5_, NiO, MoO_3_, Cu_2_O, Co_3_O_4_, CeO_2_, Bi_2_O_3_, Al_2_O_3_, etc., are also used to improve the performance of the bulk g-C_3_N_4_ [325,326,327,328,329,330,331,332,333,334,335,336,337,338,339,340,341,342,343,344,345]. The g-C_3_N_4_-based heterojunctions can be modified by combining with several metals or doping with various agents [346,347,348]. Cu is a conventional metal using for the improvement of photocatalytic performance. Zhou et al. used Cu/Al_2_O_3_/g-C_3_N_4_ for Rhodamine B degradation by H_2_O_2_ [349]. The Cu immobilized Al_2_O_3_/g-C_3_N_4_ also showed promising stability for the treatment of water pollution. Besides, copper is used as a charge separation center for hydrogen evolution, MO, and phenol solution degradation under visible light [350,351]. In addition to Cu, the noble metal Ag and Au is another metal catalyst used besides the g-C_3_N_4_-based composite [347,352,353,354,355,356]. The Ag and Au can prevent rapid recombination probability, improve the transfer of a generated electron, and enhance the visible light absorption by the surface plasmon resonance, and can be used for a wide range of applications, especially decontamination of organic pollutants. Bi, Pd, Pt, Ni, and Cd are other metal photocatalysts used for improved photocatalytic activities [357,358,359,360,361,362,363]. Some semiconductors are also used besides the metal oxide-g-C_3_N_4_ based composites. Carbon-based nanomaterials provide excellent stability, cost-effective synthesis, enhanced photogenerated electron reservoirs used in bioimaging and sensing, photocatalysis, electrocatalysis [214,336,364,365,366,367]. Xie et al. fabricated the carbon quantum dots modified with MoO_3_-g-C_3_N_4_ and demonstrated that this structure showed outstanding visible-light absorption used to degrade tetracycline (TC) from the environment [364]. Besides, among all carbon-based nanomaterials, reduced graphene oxide (rGO) has captured intensive attention [368,369]. Gong and coworkers illustrated that the charges transfer between g-C_3_N_4_ and Bi_2_Fe_4_O_9_ (BFO) was improved by using rGO. The presence of the rGO causes the separation of electrons and holes in the CB of g-C_3_N_4_ and the VB of BFO, respectively [354].

In addition to the mentioned metals and semiconductors, metal-organic frameworks (MOFs) are a novel class of porous and crystalline materials with a large surface area-to-volume ratio, high porosity, and tunable pore size that might improve the biosensor sensitivity. Since these structures are made from metal ions, clusters, and organic ligands, these materials can promote the separation and transfer of photoinduced electrons, making them a promising candidate for photocatalytic activities, such as organic pollutants degradation, water splitting, and CO_2_ reduction [370]. Cui et al. utilized the Fe-based MOFs, MIL-53(Fe), with Bi_2_O_3_ and g-C_3_N_4_ for the degradation of amino black 10B since this structure can enhance the visible light absorption range [371]. Like other heterojunctions, doping is another element to improve the performance of these composites and widen their applications [372,373,374,375,376].

Some g-C_3_N_4_-based ternary composites comprising the metal oxide compounds can promote photocatalytic activities [103,377,378,379,380]. Bismuth complex oxides are among the most efficient catalysts with layered structures beside the g-C_3_N_4_-based composites. Among various Bi-based compounds, Bi_2_O_3_ based catalysts have drawn significant attention in the different areas [381,382]. It illustrated that CuO_2_/Bi_2_O_3_/g-C_3_N_4_ nanocomposite reveals improved photocatalytic activities for decomposing of 2,4-dichlorophenol under visible light [381]. In another research work, Vattikuti et al. prepared Bi_2_O_3_/V_2_O_5_ photocatalysts anchored on the g-C_3_N_4_ nanostructure, which can be used for the phenol red (PR) pollutant degradation [383]. They also demonstrated that the efficiency of hybrid composites for the PR removal under the simulation solar light irradiation was higher than that of fabricated materials. Bi_2_O_3_/g-C_3_N_4_ heterojunctions were also conjugated with the BiPO_4_ [15]. g-C_3_N_4_/Bi_2_O_3_/BiPO_4_ hybrid exhibited the perfect photoelectric performance, which is mainly due to the high separation and photogeneration charges and can increment the oxidation/reduction rate. Bi_2_O_2_CO_3_ is another Bi contained material that shows promising photocatalytic activities [384,385]. Kumar and coworkers suggested novel magnetic g-C_3_N_4_/Bi_2_O_2_CO_3_/CoFe_2_O_4_ heterojunction with high visible light absorption for reduction of 4-nitrophenol into 4-aminophenol [386]. TiO_2_-g-C_3_N_4_ based ternary composites are widely used for photodegradation. Min et al. fabricated the Cu_2_O-TiO_2_/g-C_3_N_4_ hybrid composite for the organic dyes’ discolorations. Besides, this nanocomposite can also illustrate good performance for discolorations of RhB, MB, and MO within 3, 10, and 15 min, respectively [387]. TiO_2_-g-C_3_N_4_ composites are also anchored with CeO_2_ and metal to form g-C_3_N_4_-Me^n+1^/CeO_2_-TiO_2_ for photooxidation of toluene [388].

## 6. Application of Metal Oxide-Based g-C_3_N_4_ Nanocomposites

### 6.1. Photocatalysts

#### 6.1.1. H_2_ Generation via Water Splitting

These days, the demand for safe, efficient, and renewable energy resources instead of limited fossil fuel sources has increased among more and more people [14,184,389,390,391]. This replacement is an efficient remedy for global warming and greenhouse gases emission. The hydrogen energy content is in the range of 120 to 142 MJ kg^−1^, which is higher than that of hydrocarbon fuels. Thus, it is estimated that hydrogen will be responsible for 90% of energy production by 2080. As a result, H_2_ generation is a novel and environmentally-friendly research topic among many researchers [8,13,392,393,394,395]. One of the most recent hydrogen production techniques is the photocatalytic water splitting method via metal oxide-g-C_3_N_4_ heterojunctions by using prolific light sources [365,390,396,397,398,399,400]. For water splitting, the band position of the photocatalysts should be modified to provide the CB position more negative than the H_2_O reduction potential (0 eV vs. Normal Hydrogen Electrode (NHE)) for H_2_ generation and more positive than the H_2_O oxidation potential (1.23 eV vs. NHE) for O_2_ generation. To be more specific, the generated electrons are used for the hydrogen evolution reaction (HER) and oxygen evolution reaction (OER) via Equations (1) and (2), respectively, and the final water-splitting reaction is shown in Equation (3) [111].

Hydrogen evolution reaction (HER): 2H^+^ + 2e^−^ → H_2_, E° = 0.00 eV vs. NHE (1)

Oxygen evolution reaction (OER): H_2_O → 1/2O_2_ + 2H^+^ + 2e^−^, E°= −1.23 eV vs NHE (2)

Overall water splitting: H_2_O → H_2_ + 1/2O_2_, ΔG° = −1.23 kJ mol^–1^ (3)

where NHE is the normal hydrogen electrode.

Generally, there are three steps for each photocatalytic reaction; initially, the semiconductor absorbs light with energy equal to or higher than the bandgap to generate the electrons and holes in the valance and conductive band, respectively. Then, the photoinduced electrons and holes are moved to the surface of the semiconductor to start the reaction. Finally, the charge carriers participate in the reduction and oxidation reactions on the surface of the photocatalysts.

**Figure 17:**
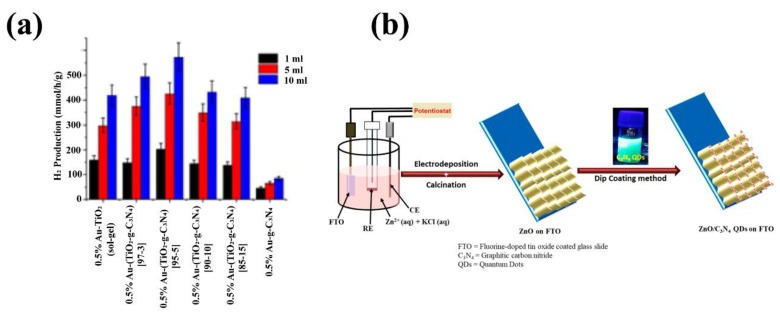
(**a**) Hydrogen production rate of different samples as a function of the amount of methanol added as a sacrificial agent under solar light irradiation, Reproduced from Reference [403]; (**b**) schematic image of the ZnO/C_3_N_4_ QDs synthesis on FTO using electrodeposition and a dip-coating method, Copyright © 2022 Elsevier [406].

Considering the band edges position of some of the metal oxide-g-C_3_N_4_ composites, which were noted previously, some heterojunctions are more anodic than that of H_2_O reduction potential to show excellent performance under visible light irradiation [111,401]. As a case in point, TiO_2_-g-C_3_N_4_ heterojunctions are widely used as an excellent photocatalytic for H_2_ evolution. Yan et al. demonstrated that the efficiency of the visible-light-induced H_2_ evolution of the binary composite comprising anatase TiO_2_ and g-C_3_N_4_ was enhanced, and this is due to the desirable photoinduced carriers’ separation [402]. In order to raise the heterojunction efficiency, other kinds of materials with various effective characteristics are also used. These materials (metals) or cocatalysts such as Ag, Au, Pt, etc., can host active sites for H^+^ reduction [403,404]. The loading Au and Ag would be significantly beneficial for this application because of their plasmonic characteristic. Marchal et al. illustrated that the optimized components ratios and contact quality in Au/(TiO_2_–g-C_3_N_4_) lead to the enhanced visible light absorption with the proper band positions for photogenerated charge carriers [403]. Besides, the presence of Au and Ag will promote the water splitting for H_2_ production by the improved charge separation rate. The H_2_ generation rate under sunlight irradiation as a function of the relative ratio of methanol as a sacrificial agent and TiO_2_/g-C_3_N_4_ is depicted in Figure 17a. It was mentioned that no H_2_ generation was observed for the Au-free g-C_3_N_4_ and TiO_2_. Furthermore, the best photocatalytic H_2_ production was observed for the 0.5 wt % Au/(TiO_2_–g-C_3_N_4_) (95/5) structure, using 1 vol% of CH_3_OH as a sacrificial agent (Figure 17a). Carbon quantum dots (CQDs) can also improve photocatalytic performance to effectively decompose H_2_O_2_ to H_2_O and O_2_. CQDs possess up-conversion fluorescence spectra, which could convert visible light to ultraviolet or near-ultraviolet light, resulting in excellent photocatalysis. Consequently, it is proven that the combination of C_3_N_4_, TiO_2_, CQDs is a great candidate for water splitting [405]. Other metal oxide-g-C_3_N_4_ based composites such as WO_3_-g-C_3_N_4_ and ZnO-g-C_3_N_4_ are used for H_2_ generation. Mahala et al. demonstrated that the prepared ZnO nanosheets decorated with g-C_3_N_4_ quantum dots composites on the fluorine-doped tin oxide (FTO) coated glass slide could be utilized as a photoanode for water splitting via PEC (Figure 17b) [406]. In another work, the effect of boron addition and carbon nitride content did increase the H_2_ evolution up to 85% compared to the bare TiO_2_, which is mainly due to charge carriers’ generation and separation [109]. It was also shown that the photoconversion efficiency of the low charge-transfer resistance of ZnO decorated with g-C_3_N_4_ is 2.3 times higher than that of pure ZnO [406]. As discussed previously, this composite had a high specific surface area, promising electronic conductivity, and excellent charge transfer interfaces and would be excellent candidates for the water splitting and H_2_ evolution [407]. Other materials with suitable properties can also be used to improve the H_2_ generation of g-C_3_N_4_–metal oxide-based composites [141,283].

In a plasmonic photocatalyst, a metal nanostructure with a size less than the wavelengths of the light, is embedded onto dielectric or semiconductor materials [408]. In such systems, the light can lead to a local electromagnetic field by generating localized surface plasmon resonance (LSPR) and hot carriers. The hot carriers move to conductive and valance bands and this phenomenon is called the LSPR sensitization [409,410]. The hot carriers can be useful for direct oxidation or reduction of chemical species. Some of the metals that showed plasmonic effects are gold (Au), silver (Ag), copper (Co), and platinum. Zhao et al. state that the LSPR of Au can enhance the light absorption and increase the number of photogenerated carriers in the Au/g-C_3_N_4_/CeO_2_ plasmonic heterojunction. The heterojunction was employed for Cr^6+^ reduction and oxytetracycline hydrochloride (OTH) catalytic degradation [411].

Metal oxide-g-C_3_N_4_ based ternary composites capture lots of attention among researchers [412]. For example, g-C_3_N_4_ (CN)/TiO_2_ (TO)/PbTiO_3_ (PTO) films were prepared by the sol-gel followed by the CVD method and were investigated for PEC water splitting [413]. Wang et al. showed the decreased resistance of the interface (R_CT_) of CN0.10/TO0.4/PTO compared to the pristine PTO and CN0.10/PTO, which facilitated the charge transfer and reduced electron-hole recombination [413]. The incident photo-to-current conversion efficiency (IPCE) was calculated to measure the PEC performance. The pristine PTO has a lower IPCE than CN0.10/PTO, which is mainly due to the higher charge recombination rate. After the TO buffer layer’s insertion, the IPCE value was drastically increased to 14.2% at 380 nm [413]. The inserted compact TiO_2_ buffer layer provided the type II and Z-scheme interfaces between PTO, TO, and CN and promoted the PEC performance with an improved current density of −68.5 μA cm^−2^ at 0 V versus Ag/Ag-Cl electrode. The high performance is mainly due to the high photogenerated ability of g-C_3_N_4_/TiO_2_ heterojunction. BiVO_4_ is another cost-effective compound with a proper bandgap (2.4 eV) to enhance the PEC water splitting [184]. Like PbTiO_3_, BiVO_4_ can also improve the photocurrent density of the g-C_3_N_4_@ZnO/BiVO_4_ heterojunction to the 0.65 mA cm^−2^ at 1.23 V versus Ag/Ag-Cl electrode.

Table 2 provides some other research activities on the water-splitting application of the g-C_3_N_4_–metal oxide-based composites.

#### 6.1.2. CO_2_ Reduction

CO_2_ emission is one of the leading environmental problems causing by fossil fuel consumption and results in a temperature rise of the earth’s surface. Photocatalytic CO_2_ reduction is a green method to deal with this problem for two reasons [111]. Not only CO_2_ reduction reduces the CO_2_ emission, but it also solves the future energy demands by producing energy fuels such as CH_4_, CH_3_OH, etc. Photocatalytic CO_2_ conversion is largely achieved by different metal oxide-g-C_3_N_4_ based systems, as they have desirable band edges positions. Equations (4)–(7) are chemical reactions for CO_2_ conversion to other solar fuels.

CO_2_ (g) + 2 H^+^ + 2 e^−^ → CO +H_2_O   E° = −0.53 eV vs. NHE at pH 7 (4)

CO_2_ (g) + 2 H^+^ + 2 e^−^ → HCOOH   E° = −0.61 eV vs. NHE at pH 7 (5)

CO_2_ (g) + 6 H^+^ + 6 e^−^ → CH_3_OH + H_2_O   E° = −0.38 eV vs. NHE at pH 7 (6)

CO_2_ (g) + 4 H^+^ + 4 e^−^ → HCHO + H_2_O   E° = −0.48 eV vs. NHE at pH 7 (7)

**Figure 18:**
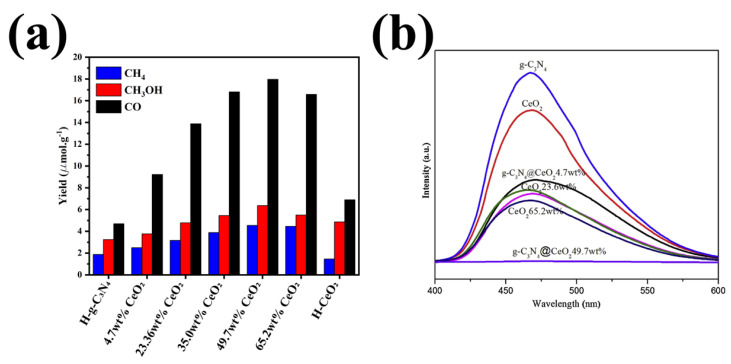
(**a**) Hydrocarbon generation of hollow g-C_3_N_4_ (H-g-C_3_N_4_), hollow CeO_2_ (H-CeO_2_), and g-C_3_N_4_@CeO_2_ for 4 h illumination; (**b**) photoluminescence emission spectra of H-g-C_3_N_4_, H-CeO_2_ references, and g-C_3_N_4_@CeO_2_ heterojunction with different ratios. Copyright © 2022 Elsevier [435].

ZnO and TiO_2_ are widely used as the g-C_3_N_4_-based composites for CO_2_ conversion [429,430]. Wang et al. designed a photocatalyst comprising TiO_2_ and g-C_3_N_4_ using ball milling and calcination. The heterostructure between TiO_2_ and C_3_N_4_ leads to a low charge recombination rate, and high separation, resulting in the high CH_4_ and CO evolution yields of 72.2 and 56.2 μmol g^−1^ are obtained [431]. In another research, Nb-TiO_2_/g-C_3_N_4_ Z-scheme heterojunctions were investigated and showed that the 50Nb-TiO_2_/50g-C_3_N_4_ composition was the best photocatalysts with high carrier separation ability for the reduction of CO_2_ [432]. The existence of electrons and holes in the CB of the g-C_3_N_4_ and VB of Nb-TiO_2_, respectively, makes the Nb-TiO_2_/g-C_3_N_4_ system a potential candidate for reducing of CO_2_ into CH_4_ and CO and HCOOH. Guo and coworkers were thermally deposited g-C_3_N_4_ onto the porous ZnO nanosheets by two-step calcination and demonstrated that the ZnO porous nanosheets @ g-C_3_N_4_-0.4 showed the highest CO_2_ conversion efficiency [433]. Not only this composite suppressed the photoinduced electron recombination and facilitated the carrier transfer, but also the CO_2_ chemosorption increased in this composite since the increasing defect vacancies formed on the porous ZnO nanosheets. Shen et al. demonstrated 3-ZnO/g-C_3_N_4_ (3 is the mass ratio of ZnO) has a high photocatalytic activity for CO_2_ reduction to CO and CH_4_ [434]. This experiment showed an insignificant decrease in photocatalytic activities, which indicates ZnO-g-C_3_N_4_ had high photocatalytic stability. A similar analysis has been performed on the hydrocarbon generation rate with hollow g-C_3_N_4_, hollow CeO_2_, which is shown in Figure 18a [435]. The fast kinetics of CO reduction results in a higher CO evolution rate compared to the CH_4_ and CH_3_OH. The highest yield obtains for g-C_3_N_4_@ 49.7 wt % CeO_2_. Increasing amounts of CeO_2_ in the g-C_3_N_4_@CeO_2_ composites will decrease the PL intensity at about 460 nm, meaning that the lower electrons and holes recombination and enhanced charge separation (Figure 18b) [435].

Other researchers also work on the CO_2_ reduction of g-C_3_N_4_–metal oxide-based photocatalysts, which is listed in Table 3.

#### 6.1.3. Photodegradation of Organic Pollutants

The development of an increased number of dye-related industries such as textile, food, and furniture manufacturing leads to severe environmental problems [445,446]. In addition to the negative aesthetic impact on water sources, the chemical oxygen demand (COD) in wastewater will be increased in the presence of organic dyes. Various methods, such as coagulation, adsorption, and membrane separation, have been used to eliminate organic dye from effluents, which only reclaim organic dyes from the wastewater liquid phase to the solid phase, creating secondary pollutants in the environment. These techniques are also a significant threat to living organisms. As a result, a metal oxide semiconductor has been widely used for the degradation of organic dyes. Photocatalysis in which most of the metal oxide can eliminate organic dyes by degradation and transfection them into particles, using solar energy for activation of the reaction. The related equations for dye degradation of the metal oxide-g-C_3_N_4_ are shown in Equations (8)–(11) [447]:

(g-C_3_N_4_+Metal Oxide) + hυ → e_CB_^−^ + h_VB_^+^ (8)

O_2_^−^ + 2e_CB_^−^ + 2H^+^ → OH^•^ + OH^−^ (9)

h_VB_^+^ + H_2_O → H^+^ + OH^•^ (10)

Organic pollutant + OH^•^ → CO_2_ + H_2_O (11)

**Figure 19:**
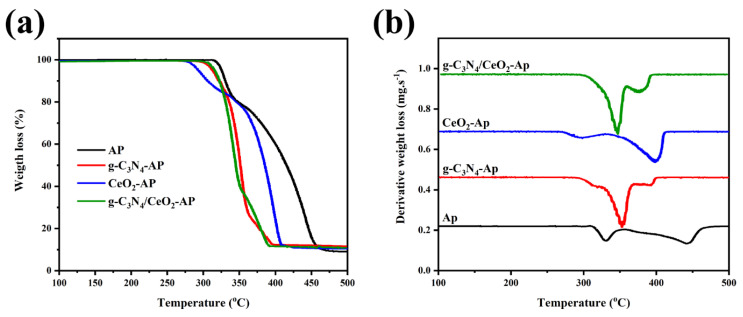
(**a**) TG and (**b**) DTG curves of pure ammonium perchlorate (AP), AP coupled with g-C_3_N_4_, CeO_2_, and g-C_3_N_4_/CeO_2_ heterojunction, Copyright © 2022 Elsevier [460].

Researchers have suggested several metal oxide semiconductors that can be used in the g-C_3_N_4_-based heterojunctions. There are increasing numbers of studies showing the degradation capability of TiO_2_, ZnO, WO_3_, Bi_2_O_3_, CeO_2_, etc. [100,101,113,116,448,449,450,451]. Zada et al. investigated the photodegrading of 2,4-dichlorophenol (2,4-DCP) and bisphenol A (BPA) over Au-(TiO_2_/g-C_3_N_4_) nanocomposites. As mentioned above, the excellent photocatalytic of the nanocomposites containing Au is mainly due to the SPR of decorated Au [131]. The structure revealed 46% and 37% for 2,4-DCP and BPA degradation, which is 5.11 and 3.1 times larger than the bulk g-C_3_N_4_ in water under visible-light irradiation, respectively [131]. In another research, the synthesized ZnO@g-C_3_N_4_ exhibited an enhanced photocatalytic activity to degrade tetracycline (TC) under visible-light irradiation, which is 2.77 and 1.51 fold more than the photocatalytic ability of pure g-C_3_N_4_ and ZnO [452]. The improved degradation was due to the enhanced transference of charge carriers and reduced charge recombination in the presence of the generated reactive oxygen species (ROS). The pharmaceuticals contaminants have hazardous impacts on human health and environmental biodiversity. Zhu and coworkers fabricated WO_3_-g-C_3_N_4_ composites for the photocatalytic degradation of one of the most well-known antibiotics, sulfamethoxazole (SMX), under visible light irradiation [453]. It is also revealed that the presence of RGO besides WO_3_-g-C_3_N_4_ heterojunctions promoted the degradation rate of ciprofloxacin (CIP) nearly twice as compared to the WO_3_-g-C_3_N_4_ structure [454]. In addition, H_2_O_2_ can raise the photocatalytic activities by the hydroxyl radicals’ productions from the degradation of a natural organic matter up to 71% for 5 h [455]. Shafawi et al. prepared Bi_2_O_3_ particles decorated on porous g-C_3_N_4_ sheets by impregnation method [456]. 1 g/L of the composite containing g-C_3_N_4_ with 9 wt % Bi_2_O_3_ at 10 ppm reactive black 5 (RB 5) at pH = 5.7 demonstrated 84% degradation efficiency under UV-vis light for 120 min. The synergistic effects between g-C_3_N_4_ and CeO_2_ provides higher catalytic activities compared to the bare ones [457,458,459]. The catalytic effects of the g-C_3_N_4_/CeO_2_ composite, bare g-C_3_N_4_, and CeO_2_ on the thermal decomposition of ammonium perchlorate (AP) were analyzed by using TGA and DTA characterization [460]. Two weight-loss regions from 25 °C to 500 °C, which were similar to the weight loss steps of AP in the absence of catalyst, were observed in Figure 19a,b. The weight loss decomposition temperatures of AP in the presence of the pure g-C_3_N_4_, CeO_2_, and g-C_3_N_4_/CeO_2_ were 53.6 °C, 47.6 °C and 74.6 °C, respectively (Figure 19a). It is also noticeable from Figure 19b that the AP thermal decomposition rate of g-C_3_N_4_/CeO_2_ nanocomposites was higher than that of CeO_2_ and g-C_3_N_4_. This heterojunction also shows a highly efficient for 2,4-dichlorophenol degradation [460].

Methylene Blue (MB), Methylene Orange (MO), and Rhodamine B (RhB) are the most commonly stable dyes in water at room temperature [461,462,463,464,465,466,467,468]. MB and RhB are toxic dyes that their high concentration can be so harmful to human and marine animal health. In accordance with the high resistivity of the MB and RhB in different environmental conditions, wastewater treatment is an urgent issue. Therefore, it is crucial to provide an effective and low-cost system to eliminate MB and RhB from sewage. As mentioned above, 2D/1D g-C_3_N_4_/ZnO nanocomposites reveal high stability, which retains the initial activity after repeated cycles [469]. The enhanced photocatalytic activity of g-C_3_N_4_-ZnO is likely due to the synergistic effects of photon acquisition and direct contact between organic dyes and photocatalyst [470]. To be more specific, the RhB dye degradation efficiency of this composite is 99% and 95% after one and three cycles under sunlight irradiation, respectively [471]. It is reported that the highest degradation efficiency for MB is obtained for the 30 wt % few-layer g-C_3_N_4_-ZnO nanocomposites is the best composition [472]. The higher amount of g-C_3_N_4_ can increase the electrons and holes recombination, leading to the decrease of photocatalytic activity. It is also noticeable that RhB degradation efficiency for this composite is about 2.1 times higher than that of pristine ZnO [473]. Apart from g-C_3_N_4_-ZnO heterojunction, g-C_3_N_4_-TiO_2_ heterojunction has high capability in the RhB degradation [474,475,476,477]. In these composites, ^•^O_2_^−^ played a major role while h^+^ played a minor role [478,479,480]. Li et al. prepared Ti^3+^ self-doped TiO_2_ nanoparticles/g-C_3_N_4_ heterojunctions and demonstrated that the Ti^3+^ and O defects improve the conductivity and light absorption range to the visible wavelength region. Besides, the photocatalytic activities for environmental purification of organic compounds (MB) of the Ti^3+^ self-doped TiO_2_/g-C_3_N_4_ nanostructures were improved under visible light irradiation remarkably [481]. It is proven that the photocatalytic activity of 2 wt % g-C_3_N_4_-TiO_2_ improved by 70% compared to the bare TiO_2_ [118]. The RhB degradation kinetic constant of g-C_3_N_4_-TiO_2_ heterojunction is 9 times and 25 times higher than g-C_3_N_4_ and TiO_2_, respectively [482]. The AgPO_4_/g-C_3_N_4_ was used for the degradation of organic pollutants. Z-scheme carbon nitride with AgPO_4_ and Ag nanoparticles was used for the degradation of RhB. The presence of Ag can improve visible light absorption. The prepared hybrid structure can improve the electrons holes separation and efficiency [483]. Other metal oxides can also be used in the g-C_3_N_4_ based heterojunction for organic dye degradation [398,484,485,486,487]. Bi_2_O_3_/g-C_3_N_4_ heterojunctions are the most commonly used composites for organic dye degradation [488,489]. Fan et al. mentioned that the ^•^OH radicals played the crucial roles during photocatalytic degradation of MB in 0.5- Bi_2_O_3_/g-C_3_N_4_ (0.5 is the mass of Bi_2_O_3_ in the synthetic process) Z-scheme heterojunction, while ^•^O_2_^−^, h^+^, e^−^ radicals have less contribution to this activity [490]. Compared to the pure Bi_2_O_3_ and bare g-C_3_N_4_, the Bi_2_O_3_/g-C_3_N_4_ heterojunctions showed better catalytic activity with 100% MB degradation ability in 90 min [490]. The 2% Bi_2_O_3_/g-C_3_N_4_ composites showed a degradation rate constant of 0.040 min^−1^, which is 2.5 and 1.9 fold higher than that found for bulk and nitrogen vacant 2D g-C_3_N_4_ nanosheet, respectively [491]. This composition showed excellent photocatalytic activity with a methylene green degradation efficiency of 98.7% under visible light irradiation [448]. At 30 °C, 5% CeO_2_/g-C_3_N_4_ (5% is the molar ratios of the CeO_2_/g-C_3_N_4_ samples) photocatalyst showed the best efficiency for MB degrading under visible light irradiation with the constant rate of 1.2686 min^−1^, which is 7.8-fold higher than pure g-C_3_N_4_ [492]. Some ternary composites are also used for RhB and MB dye degradation [493]. SnO_2_−ZnO quantum dots anchored on g-C_3_N_4_ nanosheets were demonstrated as a promising candidate for RhB degradation, which is 99% in 60 min under visible-light irradiation [494]. This hybrid also showed a promising potential for hydrogen production with the photocatalytic rate of 13.61 μmol g^−1^, which is 1.06 and 2.27 times higher than that of the binary ZnO/g-C_3_N_4_ hybrid and pristine g-C_3_N_4_. In similar work, ZnS quantum dots (ZNS)/SnO_2_/g-C_3_N_4_ ternary nanocomposites were synthesized via solid-state calcination [495]. The high bandgap of SnO_2_ and ZnS QDs leads to the reduction of the recombination rate and increases the separation of the generated carriers in the g-C_3_N_4_ [495]. The lower conduction band edge position of the SnO_2_, which is lower than others compel electrons to accumulate in this state. The position of the electrons and holes helped in improving the photocatalytic performance and enhancing stability. As a result, the transferred electrons can act as a suitable reductant and react with adsorbed O_2_ to produce superoxide radicals (O_2_^−^). On the other side, the holes in the valence band of g-C_3_N_4_ can directly oxidize the pollutants to degraded products since these holes have strong oxidizing power [495]. g-C_3_N_4_–metal oxide heterojunction is used to degrade different elements such as Hg, Cr (VI) [136,496,497], and different toxic and harmful materials such as atrazine (ATZ), chloramphenicol, ciprofloxacin (CIP), and etc. [197,454,498,499,500,501,502]. There are several research activities on the photodegradation applications of g-C_3_N_4_–metal oxide-based heterojunctions which are listed in Table 4.

### 6.2. Sensors

**Figure 20:**
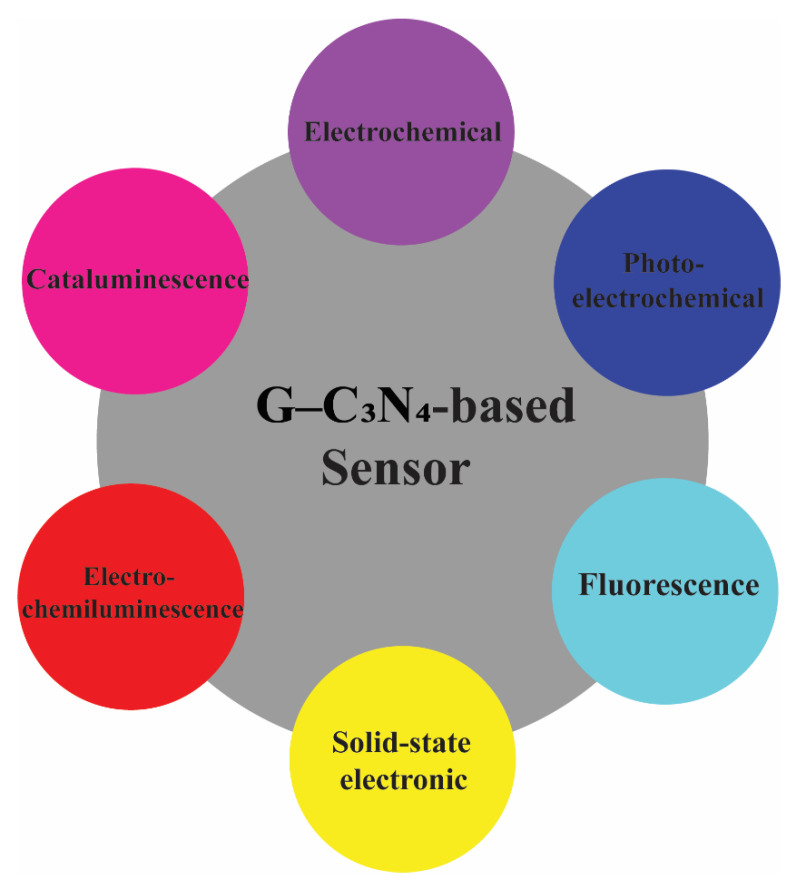
Different kinds of g-C_3_N_4_–metal oxide-based sensors for materials detection.

Some benefits, such as high sensitivity to analytes, rapid response to external stimulations, excellent fluorescence quenching abilities, light and electricity conversion properties, biocompatibility, and high stability make g-C_3_N_4_ nanosheet a promising candidate as a modified electrode for sensors to detect different analytes such as glucose, hydrogen peroxide, dopamine, etc. [6,549,550,551,552,553,554]. Metal oxide semiconductors/g-C_3_N_4_ composites are widely used as gas sensors [555]. As a result, the g-C_3_N_4_ loaded with metal oxides has also revealed new types of sensors to detect different kinds of materials [556]. General schematic description in Figure 20, shows different types of g-C_3_N_4_–metal oxide sensors.

**Figure 21:**
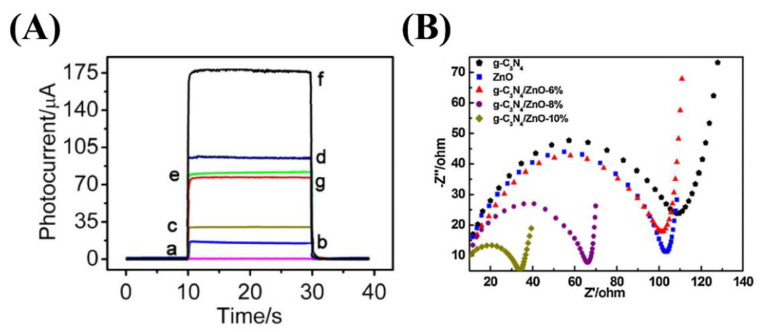
(**A**) Photocurrent responses of (a) FTO, (b) FTO/TiO_2_, (c) FTO/TiO_2_/g-C_3_N_4_, (d) FTO/TiO_2_/g-C_3_N_4_/CdS, (e) FTO/TiO_2_/g-C_3_N_4_/CdS/capture-DNA_3_/MCH, (f), FTO/TiO_2_/g-C_3_N_4_/CdS/capture-DNA_3_/MCH/DNA_2_-CdSe, (g) FTO/TiO_2_/g-C_3_N_4_/CdS/capture-DNA_3_/MCH/CdSe, Copyright © 2022 American Chemical Society [557]; (**B**) Nyquist plots of ZnO, g-C_3_N_4_/ZnO composites and g-C_3_N_4_ nanosheets electrodes, Copyright © 2022 American Chemical Society [559].

Like other applications, g-C_3_N_4_–TiO_2_ based structures are one of the most used composites for sensing applications. Li et al. demonstrated that the photoelectrochemical TiO_2_/g-C_3_N_4_/CdS platform could be employed for the ultrasensitive determination of T4 polynucleotide kinase (T4 PNK) because this composite showed significant stability and reproducibility with high selectivity [557]. The photocurrent response is demonstrated in Figure 21A. To be more specific, “trace a” shows a low photocurrent at a bare electrode. The rapidly increased photocurrent is detected in “trace b” (from 10 to 30 s) due to decrease in the rate of electron-hole recombination and the high-efficiency absorption. In the TiO_2_/g-C_3_N_4_ modified-FTO in “trace c”, the photocurrent increases to 30.7 μA since the bandgap of the g-C_3_N_4_ reduces the charge carrier recombination and facilitates the electron transmission [557]. The photocurrent increases in “trace d” (TiO_2_/g-C_3_N_4_/CdS nanocomposite-modified electrode). The photocurrent increased to 80.0 μA after attaching DNA_3_, which can capture ssDNA_2_ to the electrode and then blocking 6-mercaptohexanol (MCH) (“trace e”). For comparison, the photocurrent of CdSe QDs with or without the bio-functionalization of DNA_2_ on the electrode matrix is 180 and 78 μA, respectively [557]. In addition, “trace f” shows that DNA_2_-CdSe QDs hybridized onto the surface, the photocurrent density increases to 180.0 μA since the presence of CdSe QDs improve the light absorption efficiency. To compare, CdSe QDs also functionalized the electrode matrix without DNA_2_, and the result showed that the photocurrent density is about 78.0 μA which is similar to the “trace e” and highly lower than “trace f”. As a result, the constructed platform shows a promising candidate for ultrasensitive detection of T4 PNK activity [557]. TiO_2_ with g-C_3_N_4_ is also used for sensitive detection of protein kinase A (PKA), which is an enzyme that covalently decorates proteins with phosphate groups [558]. 

ZnO-g-C_3_N_4_ is another heterojunction used as an agent as UV-assisted gas sensors. It is shown that the ethanol (C_2_H_5_OH) sensing capability of ZnO-g-C_3_N_4_ is much higher when compared to the bare ZnO and g-C_3_N_4_. The ZnO with 8% g-C_3_N_4_ showed the best sensing performance than the others, which attributes to effective electrons and holes separation between g-C_3_N_4_ and ZnO and the UV-light catalytic effect in the room temperature [559]. The structure showed an excellent response of ethanol at room temperature, which was higher than the pure ZnO at the same condition. The arc radius of ZnO and g-C_3_N_4_/ZnO is shown in Figure 21B. The results show that adding g-C_3_N_4_ nanosheets to ZnO improves the charge transfer and reduces the electrical resistivity [559].

Sensing of NO_2_ and CH_4_ by ZnO-g-C_3_N_4_ heterojunction have also been investigated [560,561]. Hydrogen sulfide (H_2_S) is a corrosive and toxic gas, which can be generated from oil refining, mines, or petroleum fields, fuel cells, or food processing industries and may cause severe problems. As a result, eliminating H_2_S has always been a serious research topic. Zeng et al. used α-Fe_2_O_3_/g-C_3_N_4_ for H_2_S detection and showed that 5.97% α-Fe_2_O_3_ with g-C_3_N_4_ provides the best cataluminescence response [562]. The prepared α-Fe_2_O_3_/g-C_3_N_4_ composite can be used as an H_2_S gas sensor in environmental monitoring, oil refining, food processing, and so forth. Researchers have shown that g-C_3_N_4_-SnO_2_ composites can also be used as sensors for different kinds of compounds. Specifically, Zhang et al. proved that the presence of g-C_3_N_4_ beside SnO_2_ enhances the gas sensitivity and selectivity of SnO_2_ to acetic acid vapor with the reduced temperature from 230 °C to 185 °C [563]. The limit of acetic acid vapor detection is 0.1 ppm; however, the long-term stability of the prepared composite is low and should be improved. In-SnO_2_ loaded cubic mesoporous g-C_3_N_4_ will be a new method and structure to design efficient humidity sensors for monitoring indoor climatic conditions [564]. Table 5 lists the applications of g-C_3_N_4_–metal oxide heterojunction used as sensor material.

### 6.3. Bacterial Disinfection

According to different sizes, shapes, and structures of bacteria, viruses, microbes, and microalgae, the g-C_3_N_4_ has the ability to eliminate them under ultraviolet or visible light irradiation changes. Fabricating the g-C_3_N_4_ with different metal oxides is important for effective composites for water antimicrobial disinfection and microbial control. Biohazards are widely present in wastewater and contaminated water containing a variety of viruses, bacteria, fungi, etc., causing health issues in humans [30]. The pioneering work by Matsunaga et al. showed that TiO_2_ could help in inactivating bacteria such as *Escherichia coli, Lactobacillus acidophilus*, and *Saccharomyces cerevisiae* under UV light [580]. TiO_2_ suffers from low absorption of solar energy and this is due to the wide bandgap of TiO_2_, which is widely discussed in the previous parts. One method to increase TiO_2_ performance is to combine it with different semiconductors. Li et al. prepared g-C_3_N_4_-TiO_2_ using a facile hydrothermal-calcination approach with high photocatalytic bacterial inactivation ability [114]. They demonstrated that this composite could facilitate water disinfection, especially in hospital wastewaters with highly concentrated pathogenic microorganisms, using visible light. In another work, researchers investigated the excellent efficiency of this composite on the removal of the microcystis aeruginosa and Microcystin-LR [581].

In order to improve the photocatalytic activity, researchers are more likely to dope metal elements such as Ag and Cr in the g-C_3_N_4_–metal oxide structure, which can perform as an electron sink to separate the carriers. Doping these elements not only can modify the band edge positions of these structures but also, due to antimicrobial and bacterial properties of these materials, they are widely used in the g-C_3_N_4_–metal oxide-based photocatalysts [102,189]. g-C_3_N_4_/Cr-ZnO nanocomposites with superior antibacterial activity against Gram-negative (*Escherichia coli*) and Gram-positive (*Bacillus subtilis, Staphylococcus aureus*, and *Streptococcus salivarius*) were investigated [172]. In this research, the 60%g-C_3_N_4_/5%Cr-ZnO nanocomposite performs the highest antibacterial activity. Fe-SnO_2_/g-C_3_N_4_ revealed a promising sterilization performance for *Escherichia coli* and *Staphylococcus aureus* under sunlight, near-ultraviolet light, and daylight lamp [582]. The sterilization performance of this structure is mostly deserved under a daylight lamp. The magnetic silver-iron oxide nanoparticles decorated graphitic carbon nitride nanosheets showed antibacterial performance against *E. coli* bacteria [583].

**Figure 22:**
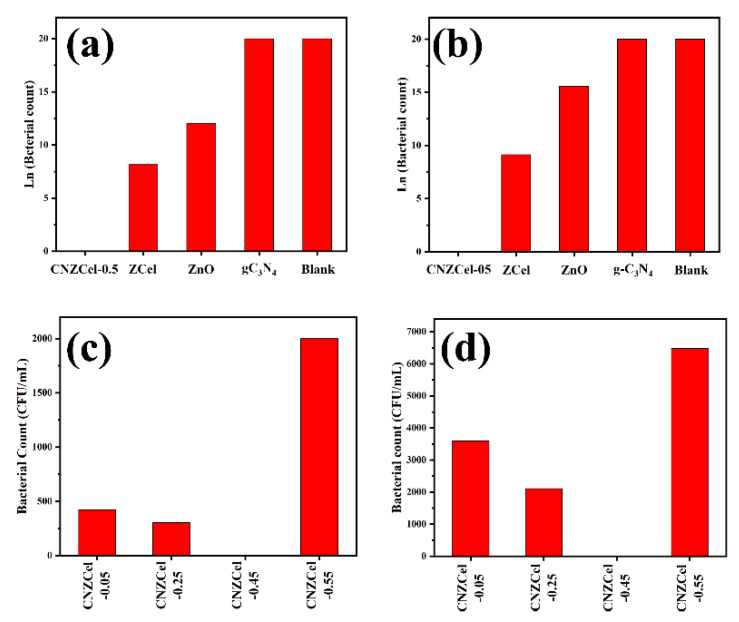
(**a**) Plots of bacterial counts of g-C_3_N_4_/ZnO/cellulose-0.45 (0.45 corresponds to the mass of g-C_3_N_4_ addition), ZnO/cellulose, ZnO, g-C_3_N_4_, and blank sample against *S. aureus* and (**b**) *E. coli*; (**c**) bacterial counts of g-C_3_N_4_/ZnO/cellulose composites against *S. aureus* and (**d**) *E. coli*. Reproduced from Reference [202].

Zhao and coworkers illustrated that g-C_3_N_4_/ZnO/cellulose (CNZCel) shows the high thermal stability of the composite and photo-excited carriers separation efficiency and decreases the recombination rate [202]. Besides, the nanocomposite revealed excellent antibacterial activity against Escherichia coli (*E. coli*) and Gram-positive bacteria *Staphylococcus aureus* (*S. aureus*). The presence of ZnO in the structure can efficiently enhance the antibacterial activities against *E. coli* and *S. aureus*. Compared to the other materials, these ternary composites demonstrated better antibacterial performance (Figure 22a,b). The effect of the g-C_3_N_4_ content on the antibacterial property against *E. coli* and *S. aureus* in this composite is also investigated, which is shown in Figure 22c,d [202]. The mechanism of antibacterial activity generates electron-hole pairs under illumination, which can provide enormous reactive oxygen species (ROS), such as ^•^O_2_^−^, ^•^OH_2_ and H_2_O_2_, needed for the inhibition of *E. coli* and *S. aureus* growth.

Table 6 mentioned some additional studies on the disinfection ability of g-C_3_N_4_–metal oxide-based nanomaterials.

### 6.4. Other Applications

Due to the specific properties of g-C_3_N_4_-based heterojunctions, these composites can be used in many other applications [150,247,585,592]. With reference to the high energy demand for electronic devices and vehicles, rechargeable lithium-ion batteries (LIBs) have captured enormous attention among many researchers [319,320,593,594,595]. Anode materials, which are a significant part of the LIBs, should have improved specific capacity, high stability. SnO_2_ is a new type of lithiophilic material with high specific capacity (1494 mAh g^−1^), low potential for Li^+^ insertion, increased number of sources, and etc. [320,593,596,597,598,599,600,601,602]; however, it suffers from significant volume expansion (∼300%) during the charge-discharge cycling, leading to fast capacity fading. SnO_2_ nanosheets with 20–25 nm thickness dispersed in the g-C_3_N_4_ showing the potential lithium storage for LIBs. SnO_2_@C_3_N_4_ nanocomposites can also be prepared by a scalable solid-state reaction [319]. Tran et al. used the hydrothermal method to grow SnO_2_ onto the graphite oxide/g-C_3_N_4_ [320]. This composite showed an excellent reversible capacity and cycling performance for lithium storage, which may attribute to the existence of g-C_3_N_4_ or graphite oxide-g-C_3_N_4_. SnO_2_@g-C_3_N_4_ based nanocomposites provide a suitable substitute for next-generation high-power and low-cost LIBs [319]. Zn_2_GeO_4_ nanoparticles demonstrate high capacity and act as spacers to prevent the g-C_3_N_4_ sheets from stacking, leading to the expanded interlayer and exposed vacancies for higher Li-ion storage. Besides, g-C_3_N_4_ layers, in turn, reduce the expansion of the particles and provide more stable solid electrolyte interphase, results in highly reversible lithium storage capacity, which is 1370 mA h g^−1^ at 200 mA g^−1^ after 140 cycles with a significant rate capability of 950 mA h g^−1^ at 2000 mA g^−1^ [595]. The SnO_2_ nanosheets with g-C_3_N_4_ can enhance lithium storage capabilities and cycling performance [593]. Other g-C_3_N_4_–metal oxide nanocomposites can also be used in the supercapacitor that can be used with batteries to mitigate the power delivery problems associated with batteries [596,597,598]. The bare g-C_3_N_4_, g-C_3_N_4_/CuO, g-C_3_N_4_/Co_3_O_4_ electrode-based device exhibited a specific capacitance of 72 F g^−1^_,_ 95 F g^−1^, and 201 F g^−1^, respectively [599]. Besides, the energy density of g-C_3_N_4_/CuO and g-C_3_N_4_/Co_3_O_4_ at the constant power density of 1 kW·kg^−1^ are 13.2 W·h·kg^−1^, and 27.9 W·h·kg^−1^, respectively. Moreover, the excellent theoretical capacities of V_2_O_5_ for the next generation supercapacitors makes the porous g-C_3_N_4_@V_2_O_5_ good candidate with a high specific capacity of about 457 Fg^−1^ at 0.5 Ag^−1^ with high cycling performance (~84% after 500 cycles) [600].

Photocatalytic nitrogen fixation, which is a clean and sustainable method for the production of NH_3_, is another application of g-C_3_N_4_-based materials. However, insignificant surface-active sites, poor stability, and high recombination rate of carriers have restricted the efficiency of g-C_3_N_4_ for nitrogen fixation activities. The carrier separation should be enhanced by doping with other elements or compositing with various metal oxides. Finally, nitrogen gas should completely adsorb on the photocatalyst for the reduction steps [603]. It is shown that the cyano group (–C☰NC) in cyano group modified g-C_3_N_4_ enhance the photocatalytic applications for N_2_ fixation up to 128 times [604].

As we discussed earlier, iodine and sulfur are atoms that can improve the carriers’ activities in the bulk g-C_3_N_4_ [605]. To be more specific, ultrathin sulfur-doped g-C_3_N_4_ porous nanosheets revealed a superior photocatalytic nitrogen fixation rate with 5.99 mM·h^−1^·g^−1^ for 4 h under simulated sunlight irradiation [606]. Zhang et al. demonstrated that B atoms change the band structures and WO_3_ enhances the photocatalytic activities. The quantum efficiency (QE) of the prepared composites is 0.71% for nitrogen fixation at 400 nm with a yield of 450.94 μmol g^−1^ h^−1^ under visible light [606]. In another work, Liu and coworkers mentioned that the phosphorus-doped 1 T-MoS_2_ as co-catalyst decorated nitrogen-doped g-C_3_N_4_ nanosheets speed up the N_2_ reduction rate to 689.76 μmol L^−1^ g^−1^·h^−1^ in deionized water under simulated sunlight irradiation, which is 2.59, 1..65, 1.47, and 1.30 times higher than that of pure g-C_3_N_4_, 1 T-MoS_2_@g-C_3_N_4_, 1 T-MoS2@N doped g-C_3_N_4_, and P doped 1 T-MoS_2_@g-C_3_N_4_, respectively. It is noteworthy to note that MoS_2_ mainly includes semiconductive 2H phase and metal octahedral 1 T phase with promising conductivity and suitable photocatalysis since this phase provides a large number of active sites on the base and edge [607]. Researchers also used metal oxides with g-C_3_N_4_-based composites to improve nitrogen fixation productivity. TiO_2_/SrTiO_3_/g-C_3_N_4_ ternary heterojunction nanofibers demonstrated N_2_ fixation value under-stimulated sunlight irradiation is 2192 μmol g^−1^ h^−1^ L^−1^, which is 1.9 and 3.3 times better than those of TiO_2_/g-C_3_N_4_ nanofibers and SrTiO_3_/g-C_3_N_4_ nanofibers, respectively [605,608]. Oxidized g-C_3_N_4_ can also enhance the chemo selective and unselective oxidative processes, utilized in organic reactions to provide synthons required in several value-added preparations [609].

It is interesting to mention that g-C_3_N_4_–metal oxide-based heterojunction can be widely used to detect of different biological materials, gasses, heavy metals, organic and inorganic materials [547,610]. g-C_3_N_4_–metal oxide-based composites can also be widely used for other applications, especially supercapacitors and desulfurization [107,611,612,613].

## 7. Conclusions, Limitations and Challenges

To fix inadequate light-harvesting of the bare g-C_3_N_4_, many researchers used doping elements to improve the efficiency, while combining g-C_3_N_4_ with other materials, such as metal oxides are another approach to deal with this issue. The investigation of g-C_3_N_4_–metal oxide-based heterojunctions with engineered bandgaps, and modified surface to enhance the abortion spectrum towards the visible-light region, reduction of the charge carrier recombination, and increasing the surface adsorption and reaction are among the main scopes of investigations of g-C_3_N_4_ based materials. Among all types of heterojunctions mentioned in the review paper, almost all g-C_3_N_4_–metal oxide-based nanocomposites have a Type II or a Z-scheme system, which have been proven to be outstanding for different applications. The applications highlighted included water splitting, CO_2_ reduction, photodegradation of organic pollutants, nitrogen fixation, catalysis, sensing, bacterial disinfection, energy storage, etc.

Several types of carbon-based nanomaterials such as carbon-based quantum dots (CDs) (carbon quantum dots (CQDs) and graphene quantum dots (GQDs)), which are discussed in our previous work, can be used as sensors [214]. One kind of carbon-based nanomaterials is g-C_3_N_4_, which is widely used to detect different cells in human biological fluids, gasses, humidity, heavy metals, etc. Nevertheless, these applications also can be enormously challenging and give plenty of room for research and development, and there are great demands for fast and accurate diagnostic methods for both clinical and commercial applications. As a result, it is suggested to develop systems for clinical use to detect different diseases outside the body, especially the newly emerging one, COVID- 19, mainly due to its high chemical stability and excellent efficiency in absorbing the light and promising photocatalytic performance. In addition, the detection of different heavy metals and ions is one of the most crucial factors for contaminant removal and water treatment. Since g-C_3_N_4_–metal oxide photocatalysts have exhibited the superior potential to detect different materials, it seems critical to extending investigations on the efficiency of these heterojunctions for detection of Cu^2+^, Hg^2+^, Cr (VI), and Ag^+^.

The need for using solar energy has increased among many researchers, as energy resources are going to be depleted. Researchers require suitable semiconductors for different catalytic reactions, such as H_2_ and O_2_ generation by water splitting, CO_2_ reduction to hydrocarbon fuels, etc. In order to have a future practical application in this area, we have to resolve some issues about the conversion of solar energy to fuel. The cocatalysis and quantum efficiency are among the most important properties for H_2_O splitting and CO_2_ reduction, which for the g-C_3_N_4_–metal oxide composites, researchers do not have enough knowledge. The enhanced photocatalytic activity of the mentioned nanocomposites in this review under visible light encourages researchers to use these heterojunctions in antibacterial and antiviral applications. Inactivation originates from direct hole oxidation and ^•^O_2_^−^, ^•^OH, and H_2_O_2_, which are produced in a reductive way, and dependent on the type of microorganisms. Because several research works have been conducted on water disinfection and microbial control in the laboratories, it is necessary to have more practical exploration on full-scale water treatment.

## Figures and Tables

**Table 1:** The electrical properties and application of doped g-C_3_N_4_.

Doping Element		E_c_	E_g_	E_v_	Application	Further Explanation	Ref
Phosphorus		−1.11 eV	2.55 eV	1.44 eV	Catalytic aromatic alcohols	The presence of P enhances the aldehyde selectivity.	[73]
		−0.33 eV	2.58 eV	2.25 eV	Photocatalytic hydrogen evolution	The g-C_3_N_4_ tube doped with P improves light absorption. The quantum efficiency of the P-doped g-C_3_N_4_ tube is 4.7 and 22.4 times higher than that of the g-C_3_N_4_ tube and bulk g-C_3_N_4_.	[74]
		−1.34 eV	2.79 eV	1.44 eV	Photocatalytic CO_2_ conversion	The P-modified g-C_3_N_4_ demonstrates the highest photocatalytic efficiency.	[75]
		−1.17 eV	2.69 eV	1.52 eV	Photocatalytic hydrogen evolution	The P-doped structure has a high efficiency in accordance with the recombination, migration, and separation of electron-hole pairs.	[76]
Sulfur		−1.04 eV	2.92 eV	1.88 eV	Photocatalytic nitrogen fixation	Sulfur enhances the adsorption and activation of N_2_ molecules of g-C_3_N_4_ porous nanosheets and uses for photocatalytic nitrogen fixation.	[77]
		−1.23 eV	2.80 eV	1.57 eV	Photocatalytic hydrogen evolution	The H_2_ generation rate of N-doped MoS_2_ and S-doped g-C_3_N_4_ is about 23 and 38 times higher than that of pure SCN and NMS with 28.8 μmol/g/h and 17.4 μmol/g/h, respectively.	[78]
		−1.3 eV	2.67 eV	1.34 eV	Photocatalytic hydrogen evolution	The BiPO_4_/S-C_3_N_4_ improves photocatalytic activity by facilitating carrier transportation.	[79]
		−1.3 eV	2.69 eV	1.39 eV	Photocatalytic bisphenol degradation	Ag–S-C_3_N_4_ enhanced the photocatalytic activity since Ag has a great electron storage ability.	[80]
		−1.32 eV	2.66 eV	1.34 eV	Photocatalytic hydrogen evolution	Sulfur promotes the photocatalytic ability of hydrogen evolution about four times higher than the bulk g-C_3_N_4_.	[81]
Oxygen		−0.88 eV	2.61 eV	1.73 eV	Photocatalytic CO_2_ reduction	The porous O-doped graphitic carbon nitride reveals enhanced photocatalytic activity.	[82]
		−0.76 eV	2.57 eV	1.84 eV	Photocatalytic hydrogen evolution	This result of the band edge value is related to the 1.1% oxygen content mass percentage.	[83]
		−0.37 eV	2.53 eV	2.15 eV		This result of the band edge value is related to the 2.3% oxygen content mass percentage.	
		−1.08 eV	2.93 eV	1.85 eV	Photocatalytic hydrogen evolution and 2,4-dinitrophenol	The oxygen dopant with Pt exhibits excellent photocatalytic hydrogen evolution in overall water splitting with 29.6 μmol/(g·h), and O-g-C_3_N_4_ NR reached up to approximately 100% removal efficiency of 2,4-dinitrophenol within 75 min.	[84]
		1.51 eV	2.70 eV	−1.19 eV	photocatalytic water splitting	This band edge value is related to CN-x = 0 (*x* refers to the quantity of citric acid (gr)).	[85]
		1.50 eV	2.62 eV	−1.16 eV		This band edge value is related to CN-0.2.	
		1.46 eV	2.52 eV	−1.06 eV		This band edge value is related to CN-0.4.	
		1.46 eV	2.49 eV	−1.03 eV		This band edge value is related to CN-0.6.	
Carbon		−1.13 eV	2.54 eV	1.41 eV	Thermal oxidation etching process	C-doped g-C_3_N_4_ improves the catalytic activity by extending the visible light absorption.	[86]
Boron		−0.8 eV	2.8 eV	2 eV	Photocatalytic Oxygen evolution, Cr(VI) reduction	This structure is used for Cr(VI) reduction and O_2_ generation simultaneously.	[87]
Nitrogen		−0.5 eV	2.4 eV	1.9 eV	Photocatalytic tetracycline degradation	This band edge value is related to the nitrogen-doped g-C_3_N_4_ used for Photocatalytic tetracycline degradation.	[88]
		−0.6 eV	2.5 eV	1.9		This band edge value is related to the nitrogen-doped g-C_3_N_4_ nanosheets.	
		−0.33 eV	1.82 eV	1.49 eV	Photocatalytic phenol degradation	N-doped g-C_3_N_4_ possesses a narrow bandgap since the N atom introduced an inter-bandgap, resulting in the redshift in the absorption UV-vis peak.	[89]
Metal	Na	−1.16 eV	2.77 eV	1.36 eV	17α-ethynylestradiol mineralization	The photostable Na doped g-C_3_N_4_ content causes the photoabsorption enhancement.	[70]
	K	−1.08 eV	2.72 eV	1.64 eV	Photocatalytic CO_2_ reduction	K content causes defects leading to improving catalytic activity by reducing the electron-hole recombination.	[90]
	Ti	−1.02 eV	2.5 eV	1.48 eV	Photocatalytic enhancement	Ti-doped g-C_3_N_4_ caused narrower bandgap and reduced carrier recombination resulting in higher absorption.	[91]
	Mn	−0.59 eV	2.56 eV	1.97 eV	Photocatalytic methylene blue degradation	The Mn-doped g-C_3_N_4_ nanoribbon reveals a great potential photocatalytic agent for water splitting coupling with MB degradation.	[92]
	Ag	-	2.60 eV	-	Photocatalytic oxidation of methylene blue	The higher Ag content leads to the lower bandgap of the structure, and a lower recombination rate is observed in the Ag-doped g-C_3_N_4_.	[93]
	Fe	−1.10 eV	2.50 eV	1.40 eV	Environmental pollution control	Fe^3+^ with nitrogen in heptazine forms a σ-π bond and can accelerate the electron-hole separation.	[94]
	Co	−0.36 eV	2.62 eV	2.26 eV	Photo-electrochemical water oxidation	Co-doped g-C_3_N_4_ reduces the electron-hole recombination rate and demonstrates promising photocurrent and electrical conductivity.	[95]
Co-doped	P, O	−0.80 eV	2.30 eV	1.50 eV	Photocatalytic fluoroquinolone antibiotics degradation	The degradation rate of enrofloxacin was 6.2 times higher for phosphorus and oxygen co-doped graphitic carbon nitride (POCN) than g-C_3_N_4_.	[96]
	P, S	-	2.6 eV	-	Photocatalytic hydrogen evolution	The high photocatalytic activity can be observed because of the synergic impact of P and S co-doping.	[97]
	B, F	-	2.72 eV	-	Photocatalytic hydrogen evolution	B, F co-doped g-C_3_N_4_ improves the charge generation and the separation efficiency.	[98]
	Na, O	-	2.72 eV	-	Photocatalytic hydrogen evolution	Na, O co-doped g-C_3_N_4_ reveals that photocatalytic H_2_ production activity was seven-fold improved by enhancing absorption of UV-vis spectra.	[99]

**Table 2:** A list of selected works on g-C_3_N_4_–metal oxide-based photocatalytic water splitting.

Photocatalyst	Type of Heterojunction	Source of Light	Highest Photocatalytic Rate	Ref
TiO_2_-g-C_3_N_4_	Type II	Asahi Spectra Hal-320 (300 mW cm^−2^) with a 420 nm cut off filter (λ > 420 nm)	3.6 μmol·h^−1^	[115]
TiO_2_-g-C_3_N_4_	Type II	Xenon lamp with a 320 nm cut off filter (λ > 320 nm)	76.25 μmol·h^−1^	[414]
C-doped TiO_2_-g-C_3_N_4_	Type II	300 W Xe lamp (PLS-SXE300) with a 420 nm cutoff filter (λ > 420 nm)	35.6 μmol·g^−1^·h^−1^	[128]
TiO_2_-g-C_3_N_4_ decorated by Co-Pi	Type II	300 W Xe lamp coupled with a monochromator	-	
TiO_2_ nanodots/g-C_3_N_4_	S-scheme	300 W Xe lamp (LANPU) with a 300 nm cutoff filter (λ > 300 nm)	The H_2_ and O_2_ evolution rate is 1318.3 and 638.7 μmol g^−1^, respectively, (roughly as same as the stoichiometric ratio of evolved H_2_ to O_2_)	[415]
ZnO-g-C_3_N_4_	Type II	PLS-SXE-300C lamp with an UV light intensity of 34 mW/cm^2^ and visible-light intensity of 158 mW/cm^2^	-	[416]
N-doped ZnO-g-C_3_N_4_	Z-scheme	PLS-SXE-300C UV lamp with a 420 nm cut off filter (λ > 420 nm)	152.7 μmol·h^−1^	[417]
g-C_3_N_4_-WO_3_	-	300-W Xe lamp (PLS-SXE300) with a 420 nm cutoff filter (λ > 420 nm)	963 μmol·g^−1^·h^−1^	[141]
O-g-C_3_N_4_/WO_3_	Z-scheme	300 W Xenon lamp with a 420 nm cutoff filter (λ > 420 nm)	15,142 μmol·g^−1^	[265]
S-Cu_2_O/g-C_3_N_4_	Z-scheme	300 W Xe lamp with a 420 nm cutoff filter (λ > 420 nm)	24.83 μmol·h^−1^	[418]
BiO_2_/g-C_3_N_4_	Type II Z-scheme	500 W Xe lamp with a 420 nm cutoff filter (λ > 420 nm)	8,542 μmol·g^−1^	[419]
g-C_3_N_4_/BiYO_3_	Type II	-	37.6 μmol·g^−1^·h^−1^	[420]
g-C_3_N_4_/La_x_Co_3-x_O_4_	-	300 W Xe lamp with a 420 nm cutoff filter (λ > 420 nm)	63.12 μmol·h^−1^	[421]
Fe_2_O_3_/g-C_3_N_4_	Z-scheme	350 W Xe lamp with a 420 nm cutoff filter (λ > 420 nm)	398.0 μmol·g^−1^·h^−1^	[210]
Mn_3_O_4_/g-C_3_N_4_	p-n heterostructure	300 W Xenon lamp with a 420 nm cutoff filter (λ > 420 nm) (PLS-SXE300D/300DUV, Beijing Perfectlight)	The H_2_ and O_2_ evolution rate is 3300 and 654 μmol g^−1^·h^−1^, respectively.	[422]
g-C_3_N_4_/Nitrogen-Doped Carbon Dots/WO_3_	-	300 W Xenon lamp with a 420 nm cutoff filter (λ > 420 nm) (CEL-HXF 300)	3.27 mmol g^–1^ h^–1^	[423]
Mn_3_O_4_/g-C_3_N_4_	-	300 W Xenon lamp source (PLS-SXE300D/300DUV)	2700 mmol·g^−1^·h^−1^	[422]
NiO/g-C_3_N_4_	Type II	Xe lamp with a 420 nm cutoff filter (λ > 420 nm)	1.41 mmol·h^−1^	[424]
In_2_O_3_/g-C_3_N_4_	Type II	300 W Xe lamp with a 420 nm cutoff filter (λ > 420 nm)	0.99 mmol·h^−1^	[425]
MoO_3-x_-g-C3N_4_	Z-scheme	300 W Xe lamp with a 420 nm cutoff filter (λ > 420 nm)	22.8 mmol·h^−1^	[426]
ZnO/Au/g-C_3_N_4_	Z-scheme	150 W Xenon arc lamp with a 420 nm cutoff filter (λ > 420 nm)	3.69 μmol h^−1^ cm^−2^	[397]
d-Ti_3_C_2_/TiO_2_/g-C_3_N_4_	-	300 W Xe lamp with a 420 nm cutoff filter (λ > 420 nm)	1.62 mmol·h^−1^g^−1^	[396]
TiO_2_/g-C_3_N_4_	Type II	450 W high-pressure mercury lamp	22.4 mol·h^−1^	[402]
TiO_2_-WO_3_-g-C_3_N_4_	-	-	286.6 mmol·h^−1^	[427]
TiO_2_/Ti_3_C_2_/g-C_3_N_4_	-	300 W Xe lamp	2592 mmol·g^−1^	[428]

**Table 3:** A list of CO_2_ reduction applications of the g-C_3_N_4_–metal oxide-based photocatalytic.

Photocatalyst	Type of Heterojunction	Source of Light	Highest Photocatalytic Rate	Ref
NiO-g-C_3_N_4_	Type II	300 W Xenon-arc lamp	4.17 μmol·g^−1^·h^−1^	[436]
g-C_3_N_4_ foam-Cu_2_O	Z-scheme	350–780 nm lamp	8.182 μmol·g^−1^·h^−1^ (CO revolution)	[437]
NiMoO_4_-g-C_3_N_4_	Z-scheme	-	7238 μmol·g^−1^·h^−1^	[438]
CeO_2_-g-C_3_N_4_	Type II	300 W of Xenon-arc lamp	0.590 μmol·h^−1^ (CO evolution)	[439]
ZnO/Au/g-C_3_N_4_	Z-scheme	300 W UV-Vis lamp	689.7 μmol/m^2^ (CO evolution)	[440]
ZnO/g-C_3_N_4_	Z-scheme	300 W xenon light source with a 420 nm cutoff filter (λ > 420 nm)	~72.24 μmol·g^−1^	[435]
TiO_2_/g-C_3_N_4_	Type II	8 W UV lamp	The highest CH_4_ and CO yields of 72.2 and 56.2 μmol g^−1^	[431]
Nb doped TiO_2_/g-C_3_N_4_	Z-scheme	1000 W Xe lamp	The CH_4_, CO, O_2_, HCOOH generation rate in the presence of 50Nb-TiO_2_/50 g-C_3_N_4_ is 562, 420, 1702, 698 μmol h^−1^ g^−1^, respectively.	[432]
ZnO/g-C_3_N_4_	Z-scheme	350 W Xe lamp	The CH_3_OH production rate was 1.32 μmol h^−1^ g^−1^	[429]
ZnO/g-C_3_N_4_	Type II	300 W xenon lamp with a 420 nm cutoff filter (λ > 420 nm)	H_2_, CH_4_, and CO production rates of 22.7 μmol·g-C_at_^−1^·h^−1^, 30.5 μmol·g-C_at_^−1^·h^−1^, and 16.8 μmol·g-C_at_^−1^·h^−1^	[433]
ZnO/g-C_3_N_4_	Type II	350 W Xe arc lamp	45.6 mol·g-C_at_^−1^·h^−1^	[441]
TiO_2_/g-C_3_N_4_	Type II	450 W Xe lamp	22.5 μmol·g^−1^ and 70 μmol·g^−1^ for CO and CH_4_ yield, respectively	[442]
g-C_3_N_4_/3D ordered microporous (3DOM)-WO_3_	Z-scheme	visible light (λ ≥ 420 nm)	48.7 μmol g^−1^ h^−1^	[443]
NiTO_3_/g-C_3_N_4_	Z-scheme	300 W xenon lamp with a 420 nm cutoff filter (λ > 420 nm)	The highest yield of CH_3_OH production is 13.74 μmol∙g^−1^∙h^−1^	[444]

**Table 4:** the photodegradation application of g-C_3_N_4_–metal oxide-based heterojunctions.

Photocatalyst	Type of Heterojunction	Source of Light	Application	Highest Photocatalytic Rate	Stability	Ref
TiO_2_-g-C_3_N_4_	Z-scheme	300 W Xenon arc lamp with a 420 nm cutoff filter (λ > 420 nm)	Degradation of Rhodamine B and tetracycline hydrochloride	The RhB removal rates for 5 layer TiO_2_, 3, 5, 7 layers g-C_3_N_4_ (0.5)/TiO_2_ were 5.1%, 17.9%, 31.2%, and 22.6%, respectively	-	[503]
P/O co-doped g-C_3_N_4_/anatase TiO_2_	Z-scheme	350 W Xenon-arc lamp as a light source with a 420 nm cutoff filter (λ > 420 nm)	Degradation of enrofloxacin	~98.5%	1 h	[504]
TiO_2_@ g-C_3_N_4_	Z-scheme	100-W xenon lamp with a 420 nm cutoff filter (λ > 420 nm)	Degradation of RhB	95.68%		[505]
MoS_2_-g-C_3_N_4_@TiO_2_	-	350 W Xenon lamp	Degrdation of Methylene Blue	97.55	1 h	[506]
g-C_3_N_4_ and polyaniline-co-modified TiO_2_	-	xenon lamp containing an optical filter	Degradation of tetrabromobisphenol A	92.42%	16 h	[507]
N-TiO_2_/O-doped N vacancy g-C_3_N_4_	Type II Z-scheme	Lamp with a 420 nm cutoff filter (λ > 420 nm)	Degradation of tetracycline hydrochloride and Cr(VI)	TC-HCl and Cr(VI) removal efficiency is 79.9% and 89.5%, respectively	-	[508]
TiO_2_@g-C_3_N_4_	Type II	300 W xenon lamp	Degradation of tetracycline antibiotic	TiO_2_@g-C_3_N_4_ photocatalyst shows the The highest tetracycline degradation rate is 2.2 mg/min, which is 2 times higher than that of TiO_2_ and 2.3 times higher than that of bulk g-C_3_N_4_.	-	[509]
TiO_2_/g-C_3_N_4_/persulfate (PS)	Type II	300 W xenon lamp with a 420 nm cutoff filter (λ > 420 nm)	Degradation of micropollutant (phenol, bisphenol A and carbamazepine	99.3%.		[510]
TiO_2_ nanowire/g-C_3_N_4_ nanosheet/graphene (G) heterostructures	-	300 W xenon lamp	Degradation of nitrobenzene	97%	4 h	[119]
Ti^3+^ and O doped TiO_2_/g-C_3_N_4_	Type II	30 W cold visible light-emitting diode	Degradation of Rhodamine B	The photodegradation reaction rate constant based on this heterojunction is 0.0356 min^−1^, which is 3.87 and 4.56 times higher than those of pristine Ti^3+^-TiO_2_ and g-C_3_N_4_, respectively.	-	[125]
TiO_2_/g-C_3_N_4_	Type II	-	Degrdation of ciprofloxacin (CIP)	68.1%	3 h	[511]
ZnO-g-C_3_N_4_	Z scheme	500 W Xe lamp, with a 420 nm cutoff filter (λ > 420 nm)	Degradation of Methylene Blue (MB)	75%	3 h	[512]
ZnO-g-C_3_N_4_	Z scheme	300 W xenon lamp	Degradation of cephalexin oxidation	98.9%	1 h	[513]
djembe-like ZnO- g-C_3_N_4_	-	150 W Xenon light sources	Degradation of MB and RhB	MB and RhB degradation efficiency are ~95% and ~97%, respectively.	50 min	[514]
WO_3_-g-C_3_N_4_		300 W Xenon lamp with a 420 nm cutoff filter (λ > 420 nm)	Degradation of tetracycline	90.54%	1 h	[515]
WO_3_-g-C_3_N_4_	Z-scheme	450 W xenon lamps	Degradation of AO7	100%	75 min	[516]
WO_3_-g-C_3_N_4_	Z-scheme	35-W Xe lamp with a radiation intensity of 1380 μW∙cm^2^	Degradation of orange G	98%	1 h	[517]
Ag-WO_3_/g-C_3_N_4_	Z-scheme	500 W Xe lamp (Beijing Bofei Technology. Co. Ltd., Beijing, China)	degradation of oxytetracycline hydrochloride	97.74	1 h	[273]
WO_3_@g-C_3_N_4_@MWCNT	Z-scheme	5 mW cm^−2^ Xe lamp (SANEI electronics-JAPAN)	Degradation of tetracycline	79.54%	2 h	[518]
g-C_3_N_4_/WO_3_	Z-scheme	-	Degradation of nitenpyram	The photocatalyst rate constant is 0.036 min^−1^ which is about 1.7 and 25 times higher than that of pure g-C_3_N_4_ and WO_3_, respectively.	-	[519]
Bi_2_O_3_- g-C_3_N_4_	Z-scheme	350 W xenon lamp with a 420 nm cutoff filter (λ > 420 nm)	Degradation of Rhodamine B	98%	80 min	[520]
Bi_2_O_3_- g-C_3_N_4_	Z-scheme	250 W Xenon lamp with a 420 nm cutoff filter (λ > 420 nm)	Degradation of tetracycline	80.2%	50 min	[521]
g-C_3_N_4_-CeO_2_	Type II	300W Xe arc lamp	Degradation of antibiotic doxycycline hydrochloride	84%	1 h	[522]
Shuttle-like g-C_3_N_4_-CeO_2_	-	300 W Xe arc lamp	Degradation of norfloxacin	88.6%	1 h	[523]
Kaolin/CeO_2_/g-C_3_N_4_	-	500 W Xenon lamp with a 420 nm cutoff filter (λ > 420 nm)	Removal of ciprofloxacin	90%	150 min	[524]
Fe_3_O_4_/CeO_2_/g-C_3_N_4_	-	300 W Xe lamp with a 420 nm cutoff filter (λ > 420 nm)	Degradation of tetracycline hydrochloride	96.63%	3 h	[525]
Au/g-C_3_N_4_ nanosheets/CeO_2_	Z-scheme	500 W Xe lamp with a 400 nm cutoff filter (λ > 400 nm)	Reduction of hexavalent chromium Oxidation of oxytetracycline hydrochloride	88.2% 95.1%	150 min	[411]
Co_3_O_4_-g-C_3_N_4_	-	250 W Xe lamp with a 420 nm cutoff filter (λ > 420 nm)	Degradation of Methyl Orange	100%	3 h	[526]
NiO-g-C_3_N_4_	Type II	500 W Xe-lamp with a 420 nm cutoff filter (λ > 420 nm)	Degradation of Methylene Blue	6.3 wt. % NiO loading shows a 2.3 times higher MB degradation rate than that of the pristine g-C_3_N_4_.	80 min	[527]
V_2_O_5_-g-C_3_N_4_	Z-scheme	500 W Xenon lamp with a 420 nm cutoff filter (λ > 420 nm)	Degradation of Congo Red and Cr (VI) reduction	-.	90 min	[528]
MoO_3_-g-C_3_N_4_	Z-scheme	150 W-Xe lamp having 1.5 AM filter which allows wavelength larger than 400 nm for the visible light-based catalytic reaction	Degradation of Rhodamine B	93%	3 h	[529]
MoO_3_-g-C_3_N_4_	Z-scheme	500W Xenon lamp	Degradation of Rhodamine B	100%	10–15 min	[530]
MoO_3_-g-C_3_N_4_	Z-scheme	visible light (λ > 420 nm)	Degradtion of tetracycline	85.9%	100 min	[531]
BiMoO_6_-g-C_3_N_4_	Z-scheme	300 W Xe lamp	Degradation of ciprofloxacin	~100	30 min	[532]
g-C_3_N_4_/SnO_2_	S-scheme	A 300 W Osram, 230 V with a 420 nm cut-off filter as used as the visible light source.	Degradation of NO	44.17%	30 min	[533]
g-C_3_N_4_-NiO	Z-scheme	30 W LED-light source	Degradation of Methyl Orange (MO)	96.8%	2 h	[461]
ZnO/g-C_3_N_4_	Type II	150 W Xe lamp	Degradation of MB and RhB	The MB and RhB degradation was ~95% and ~97%, respectively.	50 min	[514]
g-C_3_N_4_/CuO_x_	-	350W Xe lamp	Degradation of Methyl Orange (MO)	~62.5%.	70 min	[534]
WO_3_/g-C_3_N_4_	Z-scheme	300 W Xe arc lamp	Degradation of sulfamethoxazole	91.7%	4 h	[453]
g-C_3_N_4_ Nanosheets/ZnO	-	500 W Xe lamp	Photocatalytic reduction of aqueous chromium(VI)	70%	4 h	[535]
ZnO/g-C_3_N_4_	-	500 W xenon lamp	Photodegradation of Direct Blue 199 (DB)	99%	100 min	[536]
ZnO/g-C_3_N_4_	-	A 150 W xenon lamp	Degradation of tetracycline hydrochloride	97%	30 min	[452]
MoS_2_/Al_2_O_3_/g-C_3_N_4_	-	150 W tungsten halogen lamp with a 420 nm cutoff filter (λ > 420 nm)	Degradation of crystal violet (CV)	97.3%.	90 min	[537]
g-C_3_N_4_/ZnO	Z-scheme	300 W Xenon lamp cutoff filter with a 420 nm cutoff filter (λ > 420 nm	Degradation of 4-chlorophenol	~ 95%	1 h	[538]
Gd_2_O_3_ NPs@g-C_3_N_4_	-	300 W Xe lamp with a 420 nm cutoff filter (λ > 420 nm) (PLS-SXE300, Beijing Perfectlight Technology Co., Ltd., Beijing, China)	Degradation of Methyl Orange (MO) Methyl Blue (MB) Rhodamine B	72.4% 95.5% 100%	2 h	[539]
MoO_3_/g-C_3_N_4_/peroxydisulfate (PDS)	Z-scheme	350 W Xenon lamp with a 420 nm cutoff filter (λ > 420 nm)	degradation of ofloxacin (OFLX)	94.4%	2 h	[540]
ZnO/g-C_3_N_4_	Z-scheme	300 W Xe lamp	Degradation of cephalexin	98.9%	1 h	[513]
Ce_2_O/Bi_2_O_3_/g-C_3_N_4_	Z-scheme	75 W halogen lamp	Degradation of Malachite green and Rose Bengal	-	1 h	[541]
CuO/ZnO/g-C_3_N_4_	Z-scheme	400 W hallow lamp	Degradation of Methylene Blue and ammonia-nitrogen	The MB and ammonia-nitrogen degradation efficiency are ~98% in 45 min and 91% in 6 h.	45 min, and 6 h	[542]
Fe_3_O_4_/ZnO/g-C_3_N_4_	Type II	23 W white LED	Degradation of pantoprazole	97.09%.	90 min	[543]
Fe_3_O_4_/TiO_2_/g-C_3_N_4_	-	500 W Xenon Lamp with a 420 nm cutoff filter (λ > 420 nm	Degradation of Rhodamine B (RhB) and Methylene Orange (MO)	The photocatalyst shows the RhB, and MO degradation efficiency is ~96.4% in 80 min and 90% in120 min.	8 min and 120 min	[544]
g-C_3_N_4_/ZnO/TiO_2_	-	1000 W xenon lamp irradiation system equipped with a 410 nm cutoff filter under room temperature.	Degradation of p-toluenesulfonicacid (p-TSA)	100%	60 min	[545]
ZnO/Ag_2_O/g-C_3_N_4_	-	high pressure xenon short arc lamp with the light intensity of 100 mW cm^−2^	Degradation of ciprofloxacin	97.4%	48 min	[196]
WO_3_/TiO_2_@g-C_3_N_4_	-	500 W metal halide lamp	Removal of acetylsalicylate (aspirin) and methyl-theobromine (caffeine)	98%	90 min	[546]
TiO_2_@g-C_3_N_4_/Co_3_O_4_	-	300 W xenon lamp	Degradation of tetracycline (TC) and Methylene Orange (MO)	The TC (10 mg/L) and MO (25 mg/L) degradation efficiency are 91.6% and 97.8%, respectively.	1 h	[547]
g-C_3_N_4_/Bi_2_O_3_/TiO_2_	-	Xe-lamp light source with a 420 nm cutoff filter (λ > 420 nm	Degradation of Methylene Blue (MB)	The MB removal efficiency is 77.5%.	3 h	[548]
SnO_2_/ZnO@g-C_3_N_4_	-	300 W xenon lamp	Degradation of Rhodamine B dye and H_2_ production	99%	1 h	[494]
g-C_3_N_4_/NiO/ZnO/Fe_3_O_4_	-	-	removal of esomeprazole	95.05 ± 1.72%	70 min	[513]

**Table 5:** The list of applications of g-C_3_N_4_–metal oxide heterojunction used for detection.

Photocatalyst	Application	Explanation	Ref
MoO_3_-g-C_3_N_4_	Detection of Furazolidone	The high electroactive surface area (0.3788 cm^2^), as well as enhanced heterogeneous electron transfer rate (K°_eff_ = 4.91 × 10^−2^ cm·s^−1^ can detect Furazolidone with low limit of detection (LOD) (1.4 nM) with a working range of 0.01–228 μM.	[528]
Co_3_O_4_-g-C_3_N_4_	Detection of environmental phenolic hormones	This composite showed a wide detection range and a low limit of detection LOD (10^−9^ mol L^−1^)	[565]
g-C_3_N_4_-Fe_3_O_4_	Determination of Tramadol in Human Biological Fluids	LOD of this composite is ~0.1 μΜ	[566]
V_2_O_5_-g-C_3_N_4_	Detection of folic acid	The sensitivity, the LOD, and noise-to-signal ratio of the sensor is 19.02 μA mM^−1^ cm^−2^, and 0.00174 μM, and 3 (S/N = 3), respectively	[567]
g-C_3_N_4_-NiO	Detection of quercetin	The dynamic range and LOD of g-C_3_N_4_-NiO for sensing quercetin is 10 nM to 250 and 0.002 μM, respectively	[568]
Cu_2_O-g-C_3_N_4_	Humidity sensor	The response time and recovery time were 180–200 s and 5–10 s, respectively.	[569]
NiO-Co_3_O_4_-g-C_3_N_4_	Detection of tetrabromobisphenol-A	This structure showed a LOD of ~0.1 mmol L^−1^	[570]
ZnO @ g-C_3_N_4_	Detection of CCRF-CEM cells	The LOD of this compsite is ~20 cell/mL	[571]
ZnO flower-rod/g-C_3_N_4_-gold nanoparticle	Detection of carcinoembryonic antigen (CEA)	The PEC aptasensor for CEA determination is from 0.01 to 2.5 ng·mL^−1^ with detection of 1.9 pg·mL^−1^.	[572]
g-C_3_N_4_/ZnO	Ethanol sensing	Compared to the ZnO, the g-C_3_N_4_/ZnO-8% composites revealed an excellent response ~60 orders of magnitude at room temperature.	[559]
ZnO/g-C_3_N_4_	NO_2_ sensing	The response, recovery time, and LOD of ZnO/g-C_3_N_4_-10 wt % are 142, 190 s, and 38 ppb, respectively.	[561]
SnO2/g-C_3_N_4_	Ethanol gas Sensing	The composite with 7 wt % g-C_3_N_4_ content exhibited a promising gas sensing property to ethanol, which has better response and selectivity than that of the pure SnO_2_ based sensor.	[321]
g-C_3_N_4_/ZnO	CH_4_ Sensing	The higher active sites can be obtained in this structure due to the larger specific surface area leading to the great response toward 1000 ppm CH_4_.	[560]
g-C_3_N_4_-Mn_3_O_4_	H_2_S sensor	The LOD (S/N = 3, LOD) is 0.13 μg mL^−1^	[573]
α-Fe_2_O_3_/g-C_3_N_4_	H_2_S sensor	The linear detection range and detection limit of the H_2_S gas sensor were 0.88–7.01 μg mL^−1^(r = 0.998) and 0.5 μg mL^−1^(S/N = 3), respectively.	[562]
O vacancy WO_2.9_/g-C_3_N_4_	4-nitrophenol Sensing	Compared to other research works, this heterojunction showed the linear range of 0.4–100 μmol/L and a lower detection limit of 0.133 μmol/L.	[574]
b-Bi_2_O_3_/g-C_3_N_4_	Detection of chlorpyrifos	The linear detection range and detection limit of this sensor were 0.1–80 ng mL^−1^ a03 μg mL^−1^, respectively.	[575]
Fe_3_O_4_/Bi_2_O_3_/g-C_3_N_4_	Determination of Cd^2+^ and Pb^2+^	The minimum quantity for Cd^2+^ and Pb^2+^ that can be detected is 3 × 10^−9^ and 1 × 10^−9^ mol/L, respectively.	[576]
WO_3_/g-C_3_N_4_	Detection of phosmet	The limit of detection (LOD) and limit of quantification (LOQ) of this device was calculated 3.6 nM and 11.2 nM, respectively.	[577]
WO_3_/g-C_3_N_4_/MnO_2_	Detection of oxytetracycline cathodic	This sensor exhibited a wide detection range from 1 pM to 150 nM and a low detection limit of 0.1 pM.	[578]
CuO-g-C_3_N_4_	Aflatoxin B1 sensing	The limit of detection of 6.8 pg mL^−1^ for AFB1.	[579]

**Table 6:** Number of researches on the disinfection ability of the g-C_3_N_4_ metal oxide-based nanomaterials.

Photocatalyst	Source of Light	Application	Highest Photocatalytic Rate	Ref
g-C_3_N_4_/TiO_2_/Ag	Xe lamp with a 420 nm cutoff filter (λ > 420 nm)	Bactericidal efficiency against *E. coli*	the optimal bacterial inhibition of g-C_3_N_4_/TiO_2_/Ag was 84%	[102]
g-C_3_N_4_/Cu_2_O	300 W xenon lamp with 400 nm cutoff filter (λ > 400 nm)	Inactivation efficiencies of *E. coli* as well as Fusarium graminearum	g-C_3_N_4_ with 45%Cu_2_O composition revealed the inactivation efficiencies of ~7 log *E. coli*	[584]
TiO_2_/g-C_3_N_4_/SnO_2_	300 W xenon lamp with 420 nm cutoff filter (λ > 420 nm)	Bactericidal efficiency against *E. coli*	TiO_2_/g-C_3_N_4_/SnO_2_ structure showed a good *E. coli* disinfection efficiency under visible and UV irradiation, with ~−6.7 log *E. coli* and −8.2 log *E. coli*, respectively.	[314]
α-Fe_2_O_3_/CeO_2_ decorated g-C_3_N_4_	500 W Xe light with the cut-off filter of ~420 nm (λ > 420 nm)	Antibacterial activities against *S. aureus* (G+) and *E. coli* (G−) bacteria	α-Fe_2_O_3_/CeO_2_ decorated g-C_3_N_4_ exhibited perfect antibacterial to *E. coli* and *S.aureus* activity with the maximum zone of inhibition (ZOI) of 11 ± 0.5, 12 ± 0.	[585]
g-C_3_N_4_-m-Bi_2_O_4_	500 W halogen lamp with UV-cut off filter (λ > 420 nm)	Bactericidal efficiency *E. coli* and *S.aureus* bacteria	The ZOI value for E.coli and *S. aureus* bacterial strains was ~11 ± 0.5 mm and 12-13±0.5.	[586]
Ag/ZnO/g-C_3_N_4_	300 W xenon arc lamp	Bactericidal efficiency against *E. coli*	The *E. coli* disinfection efficiency of Ag/ZnO/g-C_3_N_4_ structure is ~7.4 log *E. coli*.	[190]
Ag/AgO-g-C_3_N_4_	100 W tungsten lamp	Bactericidal efficiency against *E. coli*	The 1, 2, 3, and 4 mg of catalyst showed low quantitative *E. coli* growth inhibition, which was ~17% and 65%, 97%, 99%, respectively.	[587]
Cu_2_O-g-C_3_N_4_	36 W fluorescent lamp with the cut-off filter of ~400 nm (λ > 400 nm)	Bactericidal efficiency against *B. subtilis*, *E. coli*, *S. aureus* and *P. aeruginosa*	The maximum ZOI for Cu_2_O-g-C_3_N_4_ to *B. subtilis E.coli*, *S. aureus* and *P. aeruginosa* is 22 ± 1.67, 15 ± 1.08, 11 ± 1.22, 6 ± 0.09, respectively.	[587]
g-C_3_N_4_/TiO_2_ Kaolinite	Xenon lamp with a 400 nm cut-off filter	Disinfection ability towards *S. aureus*	The disinfection efficiency of g-C_3_N_4_/TiO_2_/kaolinite is ~4.3 log cfu/mL in 5 h.	[588]
TiO_2_/g-C_3_N_4_	500 W xenon arc lamp with a 400 nm cut-off filter	Anti-fouling ability of *E. coli*	TiO_2_/g-C_3_N_4_ showed an excellent *E. coli* removal with a permeate flux of 2 times higher than that of filtration alone.	[589]
TiO_2_/g-C_3_N_4_	_-_	Bactericidal efficiency against *E. coli*	The bacterial survival rate for the TiO_2_ nanotube/g-C_3_N_4_ nanofilms is ~16%.	[590]
g-C_3_N_4_/Ag-TiO_2_	Xenon lamp	Bactericidal efficiency against both Gram-negative 18 Escherichia-coli and Gram-positive Staphylococcus-aureus	The presence of Ag in g-C_3_N_4_/Ag-TiO_2_ structure could enhance water disinfection under visible light.	[591]
g-C_3_N_4_/MoS_2_-Bi_2_O_3_	300 W xenon arc lamp	Bactericidal efficiency against *E. coli*	g-C_3_N_4_/Bi_2_O_4_ with the ratio of 1:0.5, could entirely inactivate 6-log10 cfu/mL *E. coli*.	[586]

## Data Availability

The data presented in this study are available on request from the corresponding author.
